# Intraoperative fluorescence diagnosis in the brain: a systematic review and suggestions for future standards on reporting diagnostic accuracy and clinical utility

**DOI:** 10.1007/s00701-019-04007-y

**Published:** 2019-07-30

**Authors:** Walter Stummer, Raphael Koch, Ricardo Diez Valle, David W. Roberts, Nadar Sanai, Steve Kalkanis, Constantinos G. Hadjipanayis, Eric Suero Molina

**Affiliations:** 1 Department of Neurosurgery, University Hospital of Münster, Albert-Schweitzer-Campus 1, A1, 48149 Münster, Germany; 2 Institute of Biostatistics and Clinical Research, University of Münster, Münster, Germany; 3 Department of Neurosurgery, University Clinic, Navarra, Spain; 4 Department of Neurosurgery, Dartmouth Hitchcock Medical Center, 1 Medical Center Drive, Lebanon, NH 03766 USA; 5 Division of Neurosurgical Oncology, Ivy Brain Tumor Center, Barrow Neurological Institute, Phoenix, AZ USA; 6 Department of Neurosurgery, Henry Ford Health System, Detroit, MI USA; 7 Department of Neurosurgery, Icahn School of Medicine at Mount Sinai, New York, NY USA

**Keywords:** STARD CNS, Glioma, Fluorescence guidance, Diagnostic accuracy, 5-ALA, Fluorescein

## Abstract

**Background:**

Surgery for gliomas is often confounded by difficulties in distinguishing tumor from surrounding normal brain. For better discrimination, intraoperative optical imaging methods using fluorescent dyes are currently being explored. Understandably, such methods require the demonstration of a high degree of diagnostic accuracy and clinical benefit. Currently, clinical utility is determined by tissue biopsies which are correlated to optical signals, and quantified using measures such as sensitivity, specificity, positive predictive values, and negative predictive values. In addition, surgical outcomes, such as extent of resection rates and/or survival (progression-free survival (PFS) and overall survival (OS)) have been measured. These assessments, however, potentially involve multiple biases and confounders, which have to be minimized to ensure reproducibility, generalizability and comparability of test results. Test should aim at having a high internal and external validity. The objective of this article is to analyze how diagnostic accuracy and outcomes are utilized in available studies describing intraoperative imaging and furthermore, to derive recommendations for reliable and reproducible evaluations.

**Methods:**

A review of the literature was performed for assessing the use of measures of diagnostic accuracy and outcomes of intraoperative optical imaging methods. From these data, we derive recommendations for designing and reporting future studies.

**Results:**

Available literature indicates that potential confounders and biases for reporting the diagnostic accuracy and usefulness of intraoperative optical imaging methods are seldom accounted for. Furthermore, methods for bias reduction are rarely used nor reported.

**Conclusions:**

Detailed, transparent, and uniform reporting on diagnostic accuracy of intraoperative imaging methods is necessary. In the absence of such reporting, studies will not be comparable or reproducible. Future studies should consider some of the recommendations given here.

**Electronic supplementary material:**

The online version of this article (10.1007/s00701-019-04007-y) contains supplementary material, which is available to authorized users.

## Introduction

During high-grade glioma (HGG) surgery, the infiltrative tumor margin is difficult to visualize during surgery. Inadvertent residual enhancing tumor is left behind when the surgeon relies only on differences in tissue color or texture for identifying tumor [5, 85]. For this reason, a number of surgical adjuncts or imaging technologies have been introduced during the last three decades which help the surgeon identify tumor tissue intraoperatively, such as neuronavigation, intraoperative MRI (iMRI) [9], ultrasound [37], and fluorescence guidance.

5-Aminolevulinic acid (5-ALA) has been the most widely studied agents used in fluorescence-guided surgery (FGS) of HGGs and is approved in different countries around the globe [6, 31, 61, 79, 84, 85, 100]. Off-label use of fluorescein sodium for FGS has been investigated in patient cohorts [2, 3, 18, 25, 50, 62, 64, 78], in addition to indocyanine green (ICG) [105]. Targeted fluorescence markers are under preclinical development and are slowly translating into the human setting [29, 51, 91].

Effective intraoperative fluorescence imaging relies on the assumptions that highlighted tissues truly represent tumor, that non-highlighted tissue presents normal brain, and that the targeted tissue corresponds to the pathology as delineated by preoperative imaging, e.g., MRI contrast enhancement in HGGs.

Therefore, diagnostic methods or tests proposed for proving intraoperative clinical reliability require precise evaluations to ensure they truly predict the presence or absence of tumor tissue in the brain, and provide the surgeon the information to decide (in conjunction with concerns for safety), whether further tumor resection should be performed. In addition to demonstrating a correlation between the signal of the intraoperative method and histology, the FDA requires a proof of clinical benefit for approval which does not necessarily include proof of improved survival [94]. For this purpose, detailed studies are necessary prior to regulatory approval and marketing of such methods.

At present, no detailed, consented criteria for testing the diagnostic accuracy or clinical benefit of intraoperative fluorescence imaging are available. Such criteria would allow comparability and reproducibility of methods. The comparative performance of such methods would ultimately be of interest for their future development and application.

Available guidelines on diagnostic accuracy, e.g., STARD (Standards for Reporting of Diagnostic Accuracy) [11, 71], do not address the particular requirements for intraoperative testing with its many confounders and inherent clustering of data. Although the basic principles for reporting on the accuracy of diagnostic tests are reflected herein, they have been constructed for diagnostic tests, which give *one value per patient*.

Both the Federal Drug Administration (FDA) and the European Medicines Evaluation Agency (EMA) provide guidance on the evaluation of diagnostic tests. The FDA’s “Statistical Guidance on Reporting Results from Studies Evaluating Diagnostic Tests” explicitly does not pertain to testing *multiple* samples from single patients, which would typically be the case for intraoperative tissue assessments.^1^

EMA’s “Guideline on Clinical Evaluation of Diagnostic Agents”^2^ includes recommendations for testing various stains/markers, e.g., stains used intraoperatively in detection of malignant mucosal lesions, recommending test performance to be expressed both in relation to an overall individual (per patient basis) and to lesions detected and/or organs or sites involved (per lesion or per site basis). However, EMA also does not address the numerous confounders and biases that might be encountered during testing for intraoperative fluorescence in the brain (as reviewed in the following), or other organs.

This review will discuss possible pitfalls and biases involved in testing intraoperative fluorescence, will analyze the available literature on how such biases have been handled, and make suggestions on possible guidelines for intraoperative diagnostic testing.

While this review focusses on widefield fluorescence imaging, which is in broad clinical use, it explicitly pertains to other methods of intraoperative tissue diagnosis as well, e.g., RAMAN spectroscopy, for which ample literature is available [13, 14, 24, 45, 47, 48].

### Classical evaluation of diagnostic tests

Diagnostic testing for correctly identifying disease or health prior to treatment decisions is a universal necessity in medicine. The expression “test” signifies any technique for determining whether subjects present a certain physical status or condition, e.g., if he is afflicted with a certain disease. For evaluating the accuracy of a diagnostic test, studies are used in which test results are compared to a reference or gold standard [11]. Reference standards may be laboratory examinations, imaging, pathological data, or clinical outcomes.

Resulting test values may be binary or dichotomous (with two qualities, e.g., disease or no disease), quantitative (or continuous, e.g., PSA for detecting prostate cancer, or other laboratory values), or semi-quantitative (on an ordinal scale, e.g., test strips for detecting sugar in urine [81]).

Many diagnostic tests are based on laboratory values, which ideally, if present or exceeding a certain level, will unambiguously indicate that a patient has a disease, whereas all other patients are disease-free. Due to the inevitable variability inherent to biological systems, however, such unambiguous tests are rare. Rather, there is usually some degree of overlap between test values in diseased and non-diseased patients based on the distribution of test values in either population.

Thus, with the same value of the diagnostic test, one patient may be afflicted with a condition whereas the other is healthy. Tests are therefore assessed for their *diagnostic accuracy*, i.e., the amount of agreement between the test, which is being assessed, and the reference standard which unequivocally denotes disease [11, 35].

To characterize diagnostic accuracy different measures or terms have been introduced which give information on the performance of tests, derived from the frequency with which a laboratory test or test value truly or falsely indicates disease, or misses the presence of disease, as summarized in Table 1.

**Table 1:** Diagnostic decision matrix for diagnostic accuracy

		**True disease state**	
		**Present**	**Absent**
Test result	Positive	True positive (TP) The test is positive and the subject suffers the disease	False positive (FP) The test is positive whereas the subject is healthy
	Negative	False negative (FN) The test is negative, yet the patient suffers the disease	True negative (TN) The test is negative and the subject is healthy
**Measure**		**Definition**	**Calculation**
Sensitivity		probability of a positive test result when a subject has the disease	TP/(TP + FN)
Specificity		Probability of a test being negative if a patient does not have the disease	TN/(TN + FP)
Positive predictive value		Probability that the patient has the disease if the test is positive	TP/(TP + FP)
Negative predictive value		Probability that the patient is disease free if the test is negative	TN/(TN + FN)

### Using common measures of diagnostic accuracy for intraoperative tissue diagnosis in the brain

How can traditional methods of diagnostic testing be used for testing the accuracy of intraoperative optical tissue diagnosis in brain tumor surgery?

The most important difference between studies on diagnostic tests that test for the presence of disease in the conventional sense, and intraoperative tissue diagnosis is that traditional measures were developed with *every patient generating a single measurement.* Hence, individual measurements, being from individual patients, are independent. On the other hand, studies evaluating the accuracy of intraoperative tumor diagnostics will typically be based on histology, and it will not suffice to take only one tissue sample per patient. Rather, multiple samples (clusters) will be collected per patient relating the signal of the detection method and the reference standard, histology. Multiple samples from single patients will render these clustered samples *interdependent*, an aspect which requires special consideration when assessing the accuracy of such tests.

The argument that intraoperative optical tissue diagnosis can be assessed in as simple a fashion as a laboratory test for the presence of disease therefore requires careful scrutiny. Simply adapting traditional diagnostic accuracy measurements to biopsies during brain tumor surgery is per se flawed, since samples are all collected in the diseased subject or organ *but not from healthy subjects*. In addition, biopsies in the brain will never be random, especially if the brain looks normal.

Hypothetically, the entire brain should be sampled with an infinite number of samples and analyzed to determine whether the volume of tissue detected by the optical method coincides with the volume of the tumor, i.e., to determine whether the test detects the entire tumor and the result of the test is truly dichotomous (all tumor detected or not). Needless to say, this is not an option. In practice, the sample volume is restricted by craniotomy and corticotomy to the area of the gross tumor and its immediate surroundings.

In addition, investigators are strongly limited by the number of samples they can collect, especially *in normal appearing* brain. They will have to rely on a finite number of intraoperative biopsies for histological comparisons. To do so, investigators will take samples from non-highlighted and from tissues highlighted by their diagnostic method and then examine samples histologically. Most biopsies will not be taken from normally appearing brain but rather from irregular brain tissue for obvious ethical reasons.

Thus, in contrast to the single laboratory value for a single patient (e.g., PSA for prostate cancer), trying to establish diagnostic accuracy in the brain from intraoperative tissue samples is compromised by numerous confounders and potential biases (with “bias” being defined as the result of systematic flaws or limitations in the design or conduct of a study, which distort the results [99]).

**Fig. 1:**
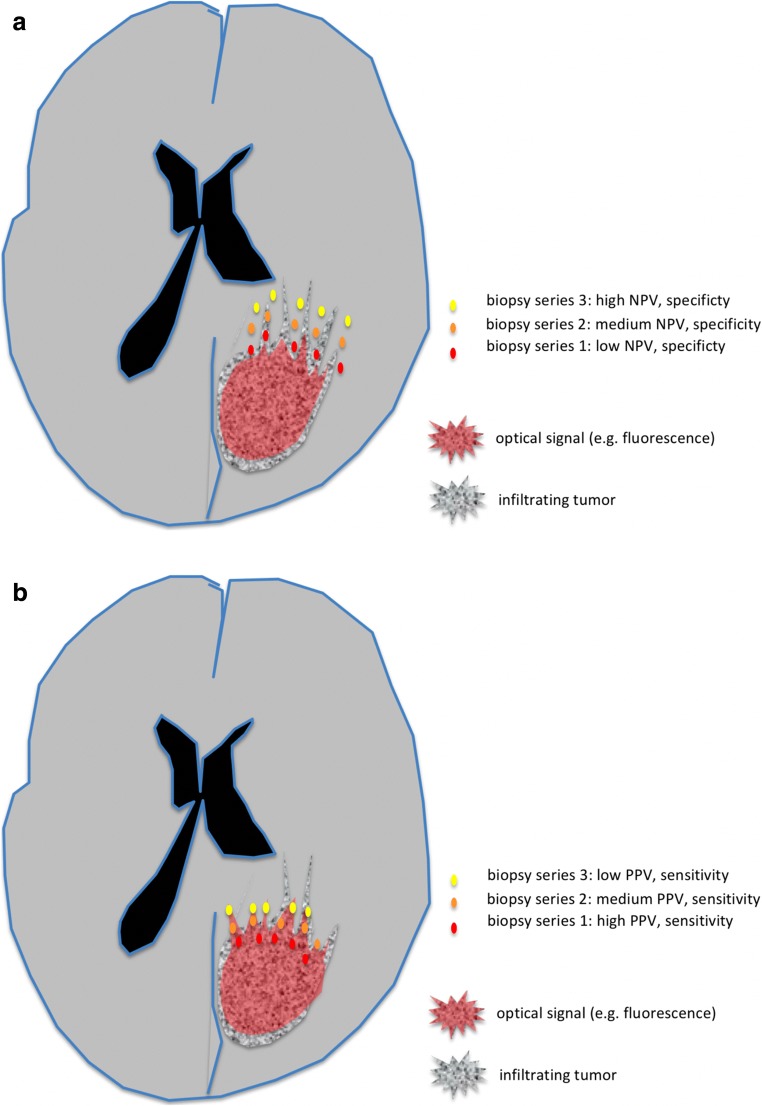
**a** Influence of tissue allocation bias type 1 on the NPV and specificity. Since gliomas are infiltrating tumors and the density of infiltrating cells will decrease rapidly with distance from the tumor bulk, the calculated NPV and specificity will be higher the further away from the tumor samples are collected because of the lower likelihood for falsely negative samples. **b** Influence of tissue allocation bias type 1 on PPV and sensitivity. The likelihood for finding falsely positive biopsies will depend on the location of biopsies. If samples are collected predominantly in the main tumor mass, the calculated PPV and sensitivity will be high. If samples are collected at the margins and the diagnostic method unreliably detects tumor, the PPV will be lower

**Fig. 2:**
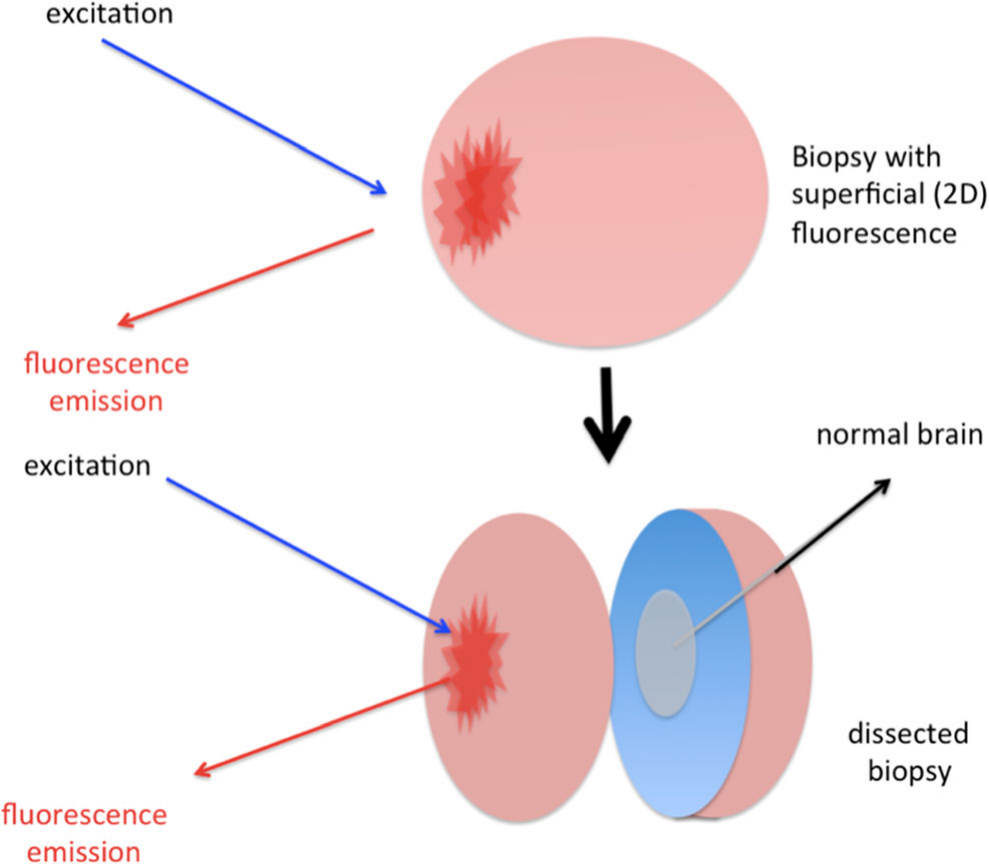
Tissue allocation bias type 3. Intraoperative optical diagnostic information is usually two-dimensional, i.e., only giving superficial information from the exposed tissue. The biopsy, on the other hand, is three-dimensional and assessment of only a part of the biopsy might miss the pathology

**Fig. 3:**
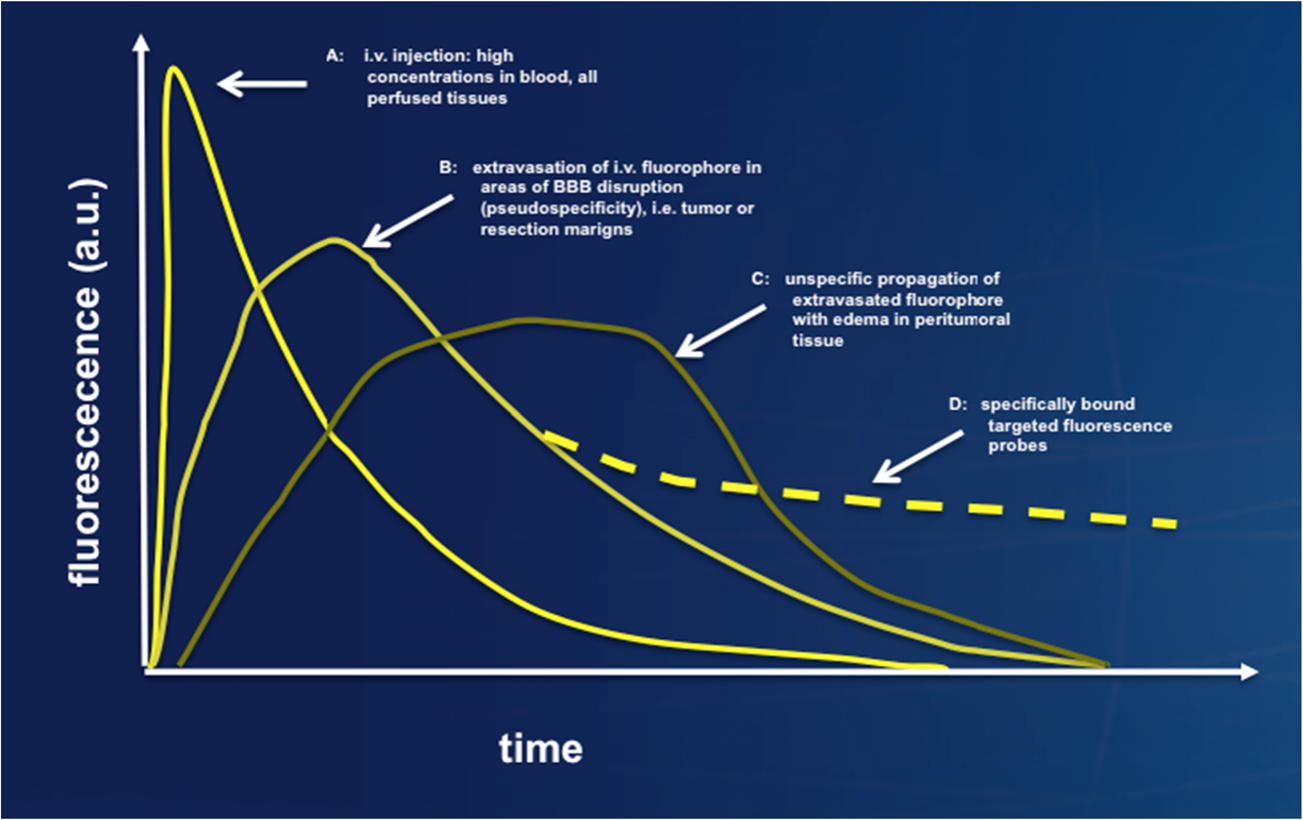
Timing and threshold bias pertinent for fluorochromes that are applied i.v. that do not have any specific tumor affinity (e.g., fluorescein sodium, Diaz et al. 2015), or expected to have selective affinity (targeted fluorochromes, e.g., APC-analoga, Swanson et al. 2015). This graph illustrates the course of fluorescence in different tissue compartments. (A) After i.v. injection, concentrations will be high in blood vessels, all perfused tissues, and will slowly abate. (B) Due to extravasation through BBB disruption within malignant tumor, pseudo-selectivity will ensue; this effect will also pertain to any areas of surgically induced BBB damage, e.g., the resection margin. (C) Meanwhile, extravasated fluorophore propagates with edema into peritumoral tissue in an unspecific manner. The apparent diagnostic accuracy will strongly depend on the definition of thresholds and on time after injection. (D) For targeted fluorochromes, selective retention can be expected after clearance from edema and plasma. These curves directly the signal-to-noise ratio, which changes over time

Another aspect which requires attention in the brain is the fact that gliomas are diffusely infiltrating tumors with cell densities tapering into surrounding brain. Tissue biopsies will not only give dichotomous results (tumor or not tumor). Rather, biopsies will reveal a variable degree of infiltration. The likelihood of finding tumor cells in biopsies, i.e., the prevalence of tumor cells, will depend on the distance of the biopsy from the main tumor mass. Traditional values for diagnostic accuracy will depend on where the biopsies are being taken, resulting in possible biases (Fig. 1a and b). Other biases allude to the way individual tissue samples are dissected for analysis (Fig. 2), the timing of surgery after application of the fluorochrome (Fig. 3), the type of staining used for identifying single tumor cells, or the number of samples taken in a certain tissue region.

An example of how biopsy numbers directly affect results of diagnostic testing is given in Table 2. Furthermore, intermixing biopsies from different patients if the numbers per patient vary, will lead to differing results depending on how these are handled (Table 3). In methods with a relevant signal-to-noise ratio that requires the creation of a threshold because of background signal, this threshold will determine the results of the test.

**Table 2:** How with a given diagnostic method, differences in the number of biopsies obtained from certain regions, based on the sampling algorithm chosen by investigator A compared to investigator B, will strongly influence the results for the measures of diagnostic accuracy

	Tumor center	Tumor margin	Normal tissue	Sens	Spec	NPV	PPV
Investigator A	*6 TP*	1 TP 2 FP	3 TN 2 FN	*0.78*	0.75	0.6	*0.78*
Investigator B	*2 TP*	1 TP 2 FP	3 TN 2 FN	*0.67*	0.66	0.66	*0.6*

In this hypothetical example, only the number of truly positive samples from the tumor center was varied, causing a relevant difference in sensitivity and positivity (italic entries).

**Table 3:** How pooling samples from different patients influences results

	TP	FP	TN	FN	Sensitivity	Specificity
Patient A	10	1	5	1	0.91	0.83
Patient B	1	1	1	1	0.50	0.50
Average measures					0.71	0.67
Pooled biopsies	11	2	6	2	0.78	0.75

The two hypothetical assessments differ only in the number of samples taken by investigators per site with a particular method

Table 4 summarizes possible biases involved in assessing the accuracy and efficacy of intraoperative diagnostic methods.

**Table 4:** Potential biases and confounders in establishing diagnostic accuracy of intraoperative optical diagnostics

Bias type	Explanation
Tissue allocation bias type A	The *location from where samples are collected relative to the signal margin* will directly influence the apparent accuracy of the diagnostic test that is being evaluated, i.e., the calculations of specificity and sensitivity, the negative predictive value (NPV) and the positive predictive value PPV (Fig. 1a and b). This is due to the fact that during intraoperative diagnostic testing in a typical, infiltrating brain tumor the prevalence of tumor cells is high in its center, whereas at the infiltrating margin the prevalence of tumor cells is lower and decreases with distance away from the tumor bulk. If samples are taken immediately beyond the margins of the highlighted tumors, the likelihood of finding unmarked, falsely negative tumor cells (FN) will be higher than if the samples are taken at a distance using the same method. This will directly affect NPV and specificity. Conversely, if marked tissue samples are taken only at the center of the tumor, the prevalence of tumor cells will be high and the rate of false positive samples low, and the calculation of PPV or sensitivity will give high values. When samples are taken at the more critical margin using the same method, it is to be expected that the rate false positive samples will be higher and the values for sensitivity and PPV lower. However, in practice PPV and sensitivity will not be as susceptible to such strong effects as the NPV and specificity, since invariably the surgeon will primarily target gross tumor, as defined by neuronavigation, ultrasound, or the optical impression under conventional illumination, and (understandably) not adjacent inconspicuous brain. *This bias can be made transparent by describing exactly the position of the biopsy relative to the signal margin.*
Tissue allocation bias type B	With methods that provide ambiguous signals, investigators are more likely to sample areas of the tumor that are judged to be abnormal with conventional illumination, e.g., by texture or color, than the inconspicuous margins, which might look like normal brain. Thus, the likelihood for true positive samples may be high with such methods despite the limitations of the method for detecting tumor at the margins. In other words, the distribution of the optical signal or tissue characteristics with conventional illumination will influence the surgeon not to adhere to a truly random biopsy regime as he will be guided to take biopsies most likely where he sees the signal or suspects tumor with conventional illumination. *To reduce this type of bias, investigators would have to predefine the biopsy site based on preoperative imaging alone, then to approach the predefined region for determining the signal of the optical method, finally to biopsy, irrespective of what is seen during surgery.*
Tissue allocation bias type C	Depending on the size of the sample, the *surface* of which is usually only visualized during surgery (due to the optical character of the methods discussed and the 2D signal returned from the tissue surface) the 3D sample might harbor different types of tissue, which in turn might confound the evaluation. This depends on how the biopsy is dissected and which fractions of the biopsy are specifically interrogated histologically (Fig. 2). Tissue allocation bias of this type has been identified as an explanation why OSNA (one step nuclei acid amplification) for detecting metastasis in sentinel lymph nodes in cancer patients compared to pathological investigations were sometimes discordant, since different parts of the same lymph nodes were tested by OSNA than by pathology (e.g., Kumagai et al. 2014). *This bias can be minimized by taking small samples or volumetrically interrogating larger samples.*
Bias from biopsy frequency	In many studies in the brain the number of typically collected tissue samples is rather low and the number per patient differ. These samples are then pooled for the final analysis of measures of diagnostic accuracy. These, however, depend on the number of samples taken in a certain brain region an entered into the calculation. Table 2 gives an example how differences in the numbers of samples in different regions in a single patient will directly influence measures of diagnostic accuracy. *This bias can be minimized by using statistical methods such as generalized linear mixed models or by keeping the number of samples for patients the same.*
Pooling samples from different patients	If a method fails to show a signal in one patient, there will be little sampling in the tumor core. In patients in whom the method works well, it is likely that more samples will be taken. Pooling these samples will skew the results and overestimate the diagnostic accuracy for single patients. Pooling biopsies from different patients without taken the dependencies of biopsies within a patient into account will, in consequence, lead to an underestimation of the variability and of the confidence limits. Also, calculating diagnostic measures per patient and then averaging over all patients will lead to biased results (Table 3). *Such uncritical pooling also ignores the interdependence of multiple samples in single patients if not statistically accounted for. Effects of such clustering should be made transparent by exactly describing the numbers of samples per patient and region. Multivariable statistical methods accounting for clustered data and potential confounders should be applied and supported by presentation of an additional patient-based analysis.*
Threshold bias in methods with significant signal-to-noise ratios	For optical methods which do not provide binary or dichotomous information (i.e., signal vs. no signal) but rather provide optical information with a wide range of values (i.e., continuous), including low level signals from normal tissue, that is, a background signal resulting in noise, the apparent discrimination between diseased tissue and normal tissue will depend on the threshold which is selected. A high threshold will decrease the likelihood of false positive samples and thus will increase PPV and specificity. A low threshold will reduce the likelihood of false negatives and will therefore increase NPV and sensitivity. For instance, spectrograpical methods will return data on a continuous scale and will be subject to this relationship, as demonstrated for 5-ALA derived porphyrin fluorescence (Valdes et al. 2011, Stummer et al. 2014). Fluorochromes, such as fluorescein sodium, which are injected i.v. and present in the plasma, will lead to a background level within normal and peritumoral tissues (Fig. 3) in a manner non-specific for tumor. *Such thresholds need to be exactly defined to minimize bias and ensure reproducibility. ROC analyses should be considered with methods that return data on a continuous scale, evaluating various thresholds.*
Timing bias	Many intraoperative imaging methods, which rely on dyes, reveal time-dependent staining of tumor tissue and also surrounding signal with a varying *signal-to-noise ratio* over time (e.g., ALA, Stummer et al. 1998,,Neira et al. 2016, Schwake et al. 2014) (Fig. 3). *Thus, results of testing for diagnostic accuracy will vary over time after administration and need to be recorded.*
Bias from methods for histological assessment	Histological assessments are clearly an important standard of truth (reference standard) for intraoperative optical testing. However, it is difficult even for the experienced neuropathologist to identify individual tumor cells based on conventional stains (e.g., H&E) only. Immunohistochemical approaches might serve to increase the likelihood of detecting tumor cells in samples, e.g., Ki67 staining, p53 or IDH1 staining, Results regarding sensitivity and specificity will vary depending on the sensitivity of neuropathological assessments and the detection of tumor cells in the peritumoral region. *Thus, the histological methods need to be reported in detail.*

#### Test result reproducibility

Multiple technical and human factors will influence the reproducibility of an intraoperative imaging test.

Technical equipment may be sensitive or insensitive in generating, detecting, and conveying the optical signal to the surgeon, and signals may vary over time due to influence by multiple factors. For example, the distance of the microscope from the illuminated cavity will determine the intensity of light reaching the cavity, which in turn will be linearly related to fluorescence intensity and may influence detection sensitivity. Typically, xenon light sources will have fluctuations and light intensity can deteriorate over time, thereby also influencing the strength of the signal. Lasers will fluctuate and will require calibration. In fluorescence, detection filters are sometimes configured to allow background light to pass. Depending on the intensity of the background signal, the test signal might be less easily detected due to background transmission of excitation light. Such effects will reduce contrast. An example for this is the Yellow 560 Zeiss, which allows a strong background signal to pass, thereby reducing the sensitivity for signal visualization. Indocyanine green (ICG) as a near-infrared (NIR) fluorochrome is invisible to the human eye and requires image processing to account for pulsation artifacts or large fluctuations in signal intensity after administration. Ambient light will interfere with tissue fluorescence in 5-ALA-induced fluorescence-guided resection. Photobleaching might play a role with all fluorochromes [86].

Also, interobserver variation will have an impact on the reproducibility when assessments are qualitative and dependent on personal judgment. An extreme example for this would be the difficulty of colorblind surgeons in differentiating red porphyrin fluorescence [67].

Some studies use technical methods for detecting specific signals from tumors, such as multiple channel spectroscopical fluorescence and/or reflectance, and generate algorithms to identify tumors based on these multiple tissue characteristics. For such methodologies, derived from training sets, with processing of multiple characteristics to give a final algorithm for tissue identification, a *validation* is required, e.g., *cross-validation or independent test cohort.* The validation is crucial to guarantee the applicability of the algorithms to data sets that differ from the particular data set used to generate the algorithm [15, 23].

#### Alternate reference standards

It is evident that histology is an important reference standard, or standard of truth. On the other hand, histology, even when a number of biopsies are obtained, will not give information about the entire tumor or the entire brain. Thus, an alternate outcome might be the completeness of tumor resection based on the intraoperative optical imaging method, as assessed by postoperative imaging, e.g., in how many cases was “complete” resection of the contrast-enhancing portion of tumor possible? In infiltrating lesions such as gliomas, it is necessary to define what should be considered as resection target on MRI. Traditionally, resection of enhancing tumor is considered as the target in high-grade glioma surgery [75, 86], whereas in low-grade gliomas, it is currently the FLAIR-weighted abnormality [57, 82]. However, tumor resection rates do not only depend on intraoperative optical methods for identifying residual tumors. The extent of resection will also be strongly influenced by *patient selection* (small, non-eloquent tumors vs. larger, eloquent tumors), the availability of intraoperative mapping/monitoring for safely performing maximal tumor resections, or the experience of the surgeon. Since these factors will differ from center to center and from surgeon to surgeon, single arm, monocentric studies will be confounded due to bias in patient selection, available resection technologies, and the surgeon. Thus, using the completeness of resection as an endpoint for evaluating intraoperative diagnostic methods will require randomized trials or prospective cohort studies, where propensity score matching or multivariable statistical methods should be applied in the analysis.

A similar argumentation pertains to outcome, i.e., survival, progression-free survival, and neurological safety. Survival has been used outside of randomized studies to indicate the benefit of a method [2].

Survival as outcome will not be directly related to the diagnostic method but rather to extent of tumor resection. Completeness of tumor resection will be under some influence of useful intraoperative optical methods, but not exclusively so, since the surgeon, who is aware of brain contrasted by a particular method may not resect tumor due to safety concerns. In the 5-ALA randomized, controlled trial in both study arms surgeons decided not to take residual visible tumor in 30% of cases due to concerns for neurological function [86]. The same limitations apply as stated for postoperative imaging. Outcomes could only be interpreted confidently when studied in prognostically balanced cohorts, which can only be achieved by randomization. However, the effects of the diagnostic method on outcome will be small since many other factors influence survival and resection rates. The outcome advantage would not be conferred by the use of the diagnostic method but “merely” by increasing the rates of more “complete” resections. Complete resections would also be observed in the control arm, and not all patients in the arm with the new diagnostic method would have complete resections for functional reasons. Thus, any effects of the intraoperative diagnostic method on outcome, for example time to progression or overall survival, would be invariably diluted and difficult to detect. The numbers needed to achieve statistical power for adequately detecting improvements of survival would therefore be very high.

#### The need for a guideline for intraoperative tissue diagnosis

Intraoperative tissue diagnosis is an expanding field. Reviews are being compiled, many of which are citing and pooling accuracy data from various publications, the accuracy data being based on classical definitions of diagnostic accuracy (e.g., sensitivity and specificity) and sometimes outcome (extent of resection and overall survival) without further consideration on how these data were determined in the original studies. Closer scrutiny reveals that rarely are possible confounders and biases accounted for or the methodology transparent enough in the original papers to allow generalization or comparison, i.e., ensuring internal and external validity.

**Fig. 4:**
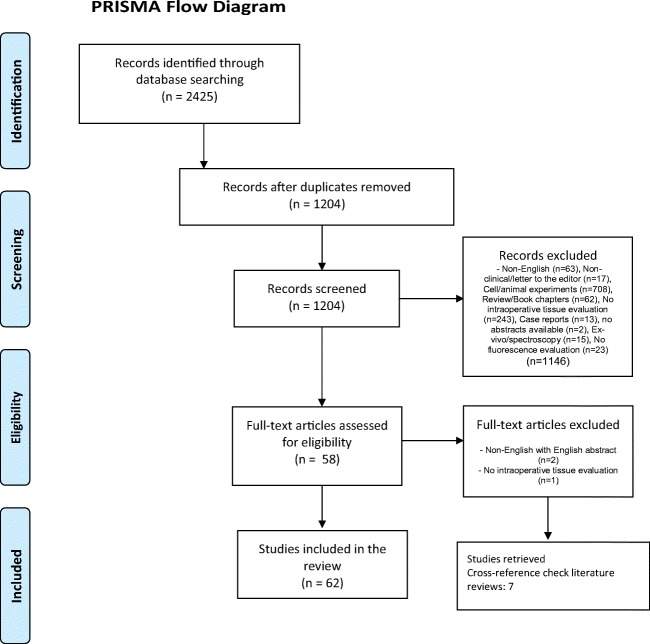
PRISMA flow diagram

For further elucidation, we reviewed all papers evaluating the use of fluorescence in brain tumor surgery, to determine how possible biases, as summarized in Table 4, were accounted for, abiding to the Preferred Reporting Item for Systematic Reviews and Meta-Analysis (PRISMA) statement [58]. MEDLINE/PubMed and Embrace data bases were interrogated for articles in English published before October 2018 with the following syntax for title and abstracts using EndNote X7 software (Thompson Reuters, Carlsbad, CA, USA): “glioma” or “gliomas” AND “fluorescence”, “fluorescence-guidance”, “fluorescence guided”, “fluorescence-guided”, “fluorophore”, “fluorochrome”, “ALA”, “5-ALA”, “5-Aminolevulinic acid”, “PPIX”, “fluorescein”, “ICG”, “indocyanin green”, “image-guided”, or “image-guidance”. The initial search delivered 2425 articles. After removing duplicates (*n* = 1221) and non-English articles (*n* = 63), all available abstracts were screened for relevance. Only articles describing clinical of fluorescence for fluorescence-guided resections of brain tumors were selected and reviewed. A cross-reference check of citations of each relevant literature review included was performed to ensure that no relevant studies were missed by the computed database search. A total of 62 studies were marked as relevant for this evaluation [1, 3, 4, 7, 8, 10, 12, 16–18, 20–22, 26–28, 32–34, 36, 38–44, 49, 50, 52, 53, 56, 59–63, 66–68, 70, 72–74, 77, 80, 85, 88–90, 92, 93, 95–98, 100, 102, 103, 105, 106] (PRISMA flow diagram: see Fig. 4).

##### Data extraction

Two authors (ESM and WS) independently extracted the following characteristics from the included studies: detection method used, study type (retro-, prospective, randomized), tumor types evaluated, outcome measures (measures of diagnostic accuracy, qualitative or quantitative outcome measures), numbers of patients, numbers of biopsies, prespecified biopsy algorithm, and whether the following sources of bias were accounted for tissue allocation biases A, B, or C, pooling of dependent and independent samples, timing, threshold (signal-to-noise ratios), types of stains used (Table 4).

#### Results of literature review

Regarding the various confounders and biases, we were able to determine the following:

- *Tissue allocation bias type A*: Only 9 of 31 studies investigating diagnostic accuracy [27, 61, 65, 74, 77, 90, 100, 101] describe biopsy locations based on the intraoperative signal margins in a reproducible way.
- *Tissue allocation bias type B*: Only 10 of 31 studies [7, 26, 32, 62, 66, 72, 74, 77, 100, 101] correlate biopsy location with tissue regions on preoperative imaging using neuronavigation.
- *Tissue allocation bias type C*: No study accounts for or gives biopsy size
- Only two studies *accommodate multiple samples per patient by using mixed models with random effects* for the individual patient [74, 96] and only one study offers a *patient-based and biopsy-based* analysis, taking care to collect the same number of biopsies in a sufficient number per patient [87].
- Only four studies have *predefined statistical analysis and sample size calculation plans* [3, 85, 87, 88].
- All studies have “blinded” pathology, but only 10 *go beyond simple H&E* staining for determining the presence of tumor cells in infiltrating tumor [1, 3, 7, 27, 38, 42, 43, 66, 77, 88], if any information is available at all.
- Only four studies use objective methods, such as spectrography, for validating the visual (subjective) optical signal [62, 90, 97] (Valdés et al. 2011: spectography; Stummer et al. 2014, spectography; Neira et al. 2016: video pixel intensities) in studies with visible fluorophores.
- *Predefined sample collection algorithms* are described in several studies [90] (e.g., Stummer et al. 2014); however, these can mostly not be considered as being reproducible if independent investigators would repeat the study.
- The *numbers of biopsies* per patient are surprisingly small in studies featuring correlations between biopsies and signal, which confounds the meaningful calculation of sensitivity or specificity of a diagnostic test (Table 5). Mean values range from 0.83 to 22 (median 4) biopsies per patient.
- Two studies do not use *reproducible reference standards* [36, 78]. The comparator is given as “helpful” or “not helpful.”
- Although administered fluorophores will have a strong time-dependent signal, *only one study* relates the *time point of biopsy collection* to the time point of fluorophore administration [62].
- Only *one randomized* study compares conventional surgery to surgery with the diagnostic method [85].

**Table 5:** Frequency of patients and biopsies in studies summarized in Table 2 (for studies with biopsies

	*N*	*n*	*n*/*N*
Mean	25.3	103	5.20
Standard deviation	21.0	85.8	4.29
Minimum	3	4	0.83
Median	21	88	4
Maximum	99	354	22

*N* number of patients in study, *n* number of biopsies per study, *n/N* number of biopsies per patient per study

**Fig. 5:**
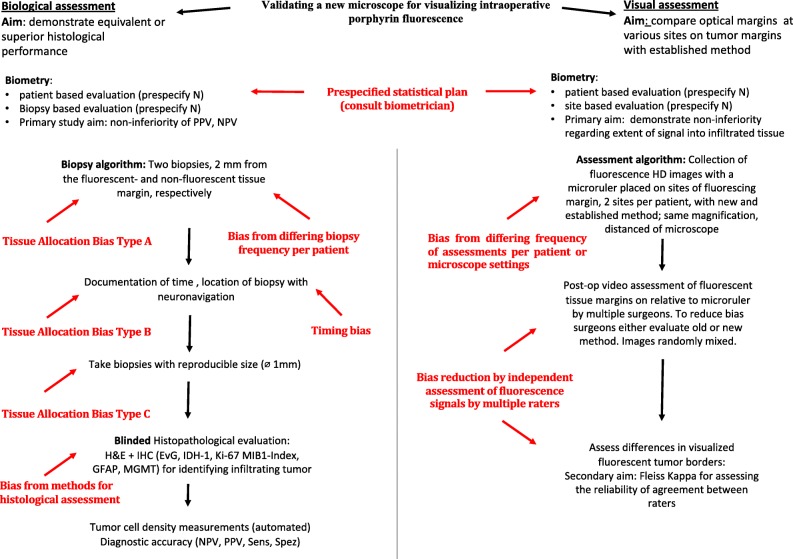
Hypothetical examples of validation algorithms of a new microscope for visualizing fluorescence in a diffusely infiltrating tumor compared to an established method. The question to be answered are: does the new method have a similar or better diagnostic accuracy, does the new method detect the same low or lower density of infiltrating cells (biological assessment, left part of the diagram), does the new method disclose the same visual margins of fluorescence (visual assessment, right). *IHC* immunhistochemistry, *EvG* Elastica van Gieson, *IDH* isocitrate dehydrogenase, *GFAP* glial fibrillary acidic protein, *MGMT* O^6^-methylguanine DNA methyltransferase

Together, most of these studies provide only minimal information necessary for reproducing results and enabling comparability or generalizability. For further illustration, we constructed a flow chart demonstrating the design of a protocol that addresses many of the biases and confounders involved in intraoperative assessments (Fig. 5).

Due to similar concerns regarding studies on the accuracy of classical tests and their “mediocre” quality [11, 71], the STARD (Standards for Reporting of Diagnostic Accuracy) initiative was born in September 2000. Reporting guidelines based on this initiative were consecutively published in several journals as open access (e.g., Bossuyt et al. [11]). It was felt that past publications with evaluations for diagnostic accuracy studies often lacked information on important aspects of design, conduct, and analysis of such studies. It was (understandably) argued that “flaws in study design can lead to biased results” [55], citing a report [55] that found diagnostic studies with specific design features to be associated with biased, optimistic estimates of diagnostic accuracy compared to studies without such deficiencies. The aim of the STARD initiative was to “to improve the quality of reporting of studies of diagnostic accuracy” with complete and accurate reporting, allowing “the reader to detect the potential for bias in the study (internal validity) and to assess the generalizability and applicability of the results (external validity)” [11].

The guidance summarized in the STARD guidelines (Electronic Supplementary Material Part 2) is pertinent and should be observed when reporting the evaluation of intraoperative diagnostic tests. However, the STARD guidelines do not address the specific requirements of intraoperative diagnostic imaging in the brain or in other organs, as they were designed for diagnostic tests where one subject gives one test value which is compared to a reference standard, in order to detect a condition of interest in that subject.

More recently the TRIPOD (Transparent Reporting of a multivariable prediction model for Individual Prognosis Or Diagnosis) guidelines were devised [19] with a similar intention of improving reporting of diagnostic models (but also of prognostic models). The TRIPOD guidelines could be pertinent in the present context, e.g., if multivariable modeling of, e.g., PPV and NPV were performed, paying attention to variables influencing these measures, such as tumor size, or location of biopsies. However, due to its more general nature, this guideline is not entirely sufficient in providing guidance for the detailed context of intraoperative imaging in neurosurgery.

Thus, for the novel and expanding field of intraoperative optical diagnosis, there is an evident need for a guideline for designing and reporting diagnostic accuracy studies.

For this purpose, we suggest expansions of the original STARD guidelines (which may be downloaded under https://www.elsevier.com/__data/promis_misc/ISSM_STARD_Checklist.pdf), as summarized in Table 6, as well as several considerations and recommendations regarding statistics, which are added in Electronic Supplementary Material 2.

**Table 6:** STARD-CNS

1. Introduction As with the STARD initiative [11], it is the aim of this guideline to help investigators improve the design and the reporting quality of diagnostic accuracy studies. STARD-CNS expands the original STARD guidelines [11] to encompass the area of intraoperative optical diagnostics with special reference to the brain and gives advice for the design of respective studies, which considers the plethora of pitfalls and biases involved in such studies. The authors feel that adherence to these recommendations will reduce the potential for inadvertent bias and to promote comparability, reproducibility and generalizability of results obtained for various intraoperative methods of optical imaging. These recommendations do not only pertain to fluorescence methods but to any methods that relate tissues identified intraoperatively to imaging and/or histology, e.g., other forms of non-optical tumor identification such as navigation per se, intraoperative MRI, ultrasound, but also to targeted fluorochromes or narrow field methods such as OCT, RAMAN, confocal imaging and others. Also, these suggestions may not only be pertinent for gliomas but might be extended to other tumor entities in the brain as well (e.g., metastasis, meningiomas, adenomas) for which intraoperative detection methods are being developed or employed. Furthermore, methods of intraoperative tissue detection are also being explored for the surgery of tumors outside the CNS, where similar considerations regarding the evaluation of such methods are justified, e.g., for mapping of sentinel lymphatic node or identification of solid tumors by near infrared fluorescence (as reviewed in Schaafsma et al. [76]).	
2. Recommendations pertaining to the design of a study • Consider a protocol with intraoperative neuronavigation and postoperative imaging for assessing the extent of the detection signal and how this relates to MRI morphology. • Consider addressing a particular tissue area first based on navigation, which relates this area to imaging data, then assessing the detection signal and finally collecting a biopsy. • In protocols containing neuronavigation for correlating tissue signal to imaging, methods should be described that compensate for the influence of brain shift • Consider histological assessment of the complete biopsy (the smallest unit of resection) and not only of a part of the biopsy • Consider expanding simple H&E histology by immunohistochemistry for better detection of infiltrating tumor cells • Consider focusing on the PPV in conjunction with the NPV (giving an exact description of *where* samples were taken in relationship to neuronavigation MR imaging). PPV is the only accuracy measurement, which does not require sampling from “normal” brain. • Consider using objective methods (e.g., spectrography) to validate subjective optical impressions. • Consider additional reference standards, i.e., extent of resection and outcome (safety, survival), apart from biopsies. • Define statistical methods for confirmatory endpoints ex ante. Involve a statistician in the planning stage (see Electronic Supplementary Material for recommendations pertaining to statistical analysis and handling of dependent and clustered samples). • If an equivalent and sufficient number of biopsies per patient cannot be collected, consider appropriate statistical methods to adjust for varying numbers of biopsies (see below). • Consider randomization to analyze the usefulness of the method for improving resection rates on MRI and outcome to achieve independence from non-therapeutic factors, such as resectability, age etc. • In studies using a method with algorithms for identifying tumor based on a specific tissue characteristic, such as with optical properties (reflection, fluorescence) with processing of multiple inputs to give a final algorithm for tissue identification, a validation cohort is required to rule out algorithms only to be valid for the particular data set used for generating the algorithm (e.g., Butte et al. [15]). _______________________________________________________________________________________________________________________ 3. Checklist for reporting, expanding the STARD Checklist [11]: Bias reduction: • What methods were used to reduce rater bias, e.g., blinded assessments by pathologists or radiologists? • Were optical signals validated by objective detection technology, i.e., spectrography? • Did multiple raters address the optical signal independently? Tissue sampling algorithms: • Describe exactly *where* tissue samples are taken and give methods for documenting the location of biopsies (e.g., neuronavigation), including the size of biopsies • Were the location and the number of biopsies taken per patient documented? • With time sensitive methods of detection (e.g., fluorochromes injected i.v.): Are the time points at which biopsies were taken described? • It is recommended that the same number of samples be taken from similarly defined locations in individual patients. How was this handled?	
Signal detection: • If the methodology employs thresholding, were the thresholds and the rationale for the thresholds exactly described? How was the background signal handled? Was ROC analysis employed for continuous data? Where values transformed? • If the methodology requires image processing, the exact procedure and settings need to be described in a reproducible way. • How was the technical equipment tested and maintained? • What factors confound signal detection and how are these handled? • Was intraobserver variability accounted for? • If algorithms for tissue detection are constructed using multiple inputs, was an independent cohort for cross-validation included? Reference standard • Describe which types of histological assessment are implemented, e.g., was immunohistochemistry used for identifying tumor cells in low density that infiltrate the brain? Which markers were assessed, e.g., Ki-67/MIB-1 staining, EGFR, GFAP, IDH1, p53, others? • If other reference standards are used (post-OP imaging, outcome, other optical imaging methods), are these exactly described? • What methods are used to ensure transparency in non-histological reference standards to allow comparability? Statistical considerations: • Was a statistician involved in the planning stage? Was a sample size calculation performed? What are the planned settings (type I error, power, assumed effects)? • What are the primary endpoints and statistical hypotheses? • What is the statistical design? • Were multiple testing procedures used for type I error control? • Were different diagnostic tools compared? Which statistical method was used? • Describe exactly how dependent data (biopsy within patient) and independent data (per patient) were handled. • Are statistical methods applied to account for the clustered data structure and differences in the number of biopsies per patient (e.g., generalized linear mixed models)? • Describe the applied statistical methods exactly and reproducibly. • Describe how missing data were handled. • Report estimates of diagnostic accuracy and measures of statistical uncertainty (e.g., sensitivity, specificity, PPV, NPV, and corresponding 95% confidence intervals). Are CI adjusted for clustered data structure? • If possible, use dichotomous outcomes for pathology and dichotomous or continuous measures for the diagnostic tool.	

## Conclusion

In conclusion, the biases and confounders involved in reliable and reproducible testing of diagnostic accuracy in methods of intraoperative imaging diagnoses are many. In this rapidly expanding field, a consensus on reporting standard is becoming necessary. If investigators do not adhere to such or similar standards, different methods or different studies using the same visualization method simply cannot be compared.

The authors propose a guideline to this end, as suggested and elucidated in Table 6 (references cited in Electronic Supplementary Material [30, 46, 54, 69, 83, 104, 107]).

## Electronic supplementary material

ESM 1 (DOCX 127 kb)

ESM 2 (DOCX 20 kb)

## Footnotes

1
http://www.fda.gov/downloads/MedicalDevices/DeviceRegulationandGuidance/GuidanceDocuments/ucm071287.pdf

2
http://www.ema.europa.eu/docs/en_GB/document_library/Scientific_guideline/2009/09/WC500003580.pdf
