# Supplementary material for: Intraoperative fluorescence diagnosis in the brain: a systematic review and suggestions for future standards on reporting diagnostic accuracy and clinical utility
Source: Acta Neurochir (Wien). 2019 Jul 30;161(10):2083–98. doi: 10.1007/s00701-019-04007-y (PMC6739423; doi:10.1007/s00701-019-04007-y)
Supplement: Supplementary file 1 — (DOCX 127 kb) [file 701_2019_4007_MOESM1_ESM.docx]

| **Publication** | **Type (optical/ video imaging), dose if and time point admistration** | **Study type**  **prospective, retrospective with predefined statistical plan (PSP)** | **Tumor histology** | **Measures of diagnostic accuracy (yes/no)**  **ROC?** | Number of patients | Number of biopsies overall/per patient | Reproducible biopsy  algorithm | Tissue Allocation Bias A accounted for reproducibly? (location rel. to signal) | Tissue Allocation Bias B accounted for reproducibly? (location relative to imaging) | Tissue Allocation Bias C accounted for? (handling of samples) **2D vs. 3D sample** | Different biopsy frequencies per patient accounted for statistically | Staining with immunhistochemistry | Pooling of dependent with independent samples;  Patient based assessment? | Timing of each biopsyrecorded rel. to application | Threshold bias (image processing or for MRI, FET imaging) accounted for (exact definition of tumor on imaging) | Alternate outcomes  quantitative | Alternate outcomes  qualitative | Randomization/case control | comments |
| --- | --- | --- | --- | --- | --- | --- | --- | --- | --- | --- | --- | --- | --- | --- | --- | --- | --- | --- | --- |
| Acerbi F, Acta Neurochir (Wien). 2015;157(8):1377-8^1^ | Visual Fluorescein, 5-10mg/kg 20% solution, after intubation before skin incision | Prospective. No PSP | GBM 19, AA 1 | Sensitivity 94, specificity 89.5% | 20 | 36 in 9 patients, 4 each | Limited (half from fluoresceing, half from non-fluorescing | n.g. | n.g. | n.g. | n.g. | Yes, GFAP, Ki 67 | Pooling, no patient bases assessment | n.g. | Not applicable | PFS, OS, resection rates, NIHSS | n.g. | no | BLUE 400 and Yellow 560; Letter with preliminary data |
| Acerbi F, et al. Acta Neurochir (Wien). 2013;155(7):1277-86.^2^ | Visual Fluorescein, 5-10mg/kg 20% solution, after intubation before skin incision | Prospective | 12 GBM. | Sensitivity 91, specificity 100% | 12; 5 for biopsies | 20 in 5 patients, 4 each | Limited (half from fluoresceing, half from non-fluorescing | n.g. | n.g. | n.g. | n.g. | GFAP, Ki 67 | yes | n.g. | Not applicable | Resection rate. NIHSS | No adverse reaction | no | BLUE 400 and Yellow 560 Possibly same patients as 1. |
| Acerbi et al. Clin Cancer Res. 2018;24(1):52-61^3^ | Visual Fluorescein, 5-10mg/kg 20% solution, after intubation before skin incision | Prospective | 44 GBM, 1 GS, 1 AA | sensitivity  80.8%, specificity79.1%. PPV 80.8% and NPV 79.1% | 46 | A total of 50 biopsies (26 in fluorescent tissue and 24 in  Non fluorescent tissue) were performed at the tumor margin in  13 patients. | Limited (“half from fluoresceing, half from non-fluorescing”) | n.g. | n.g. | n.g. | n.g. | n.g. | yes | n.g. | Not applicable | PFS; OS, resection rates, NIHSS | Adverse events | no | BLUE 400 and Yellow 560 possibly same patients as 1, 2, patient with extension into functional tissue excluded |
| Arita et al. Cancer. 2012;118(6):1619-27^4^ | Visual, 20 mg/kg 1-2 hours prior to intubatoin | Prospective, no PSP | 2 grade II, 2 grade III, 7 GBM | n.g. | 11 | 1-5, total 30 | n.g. | n.g. | Yes, neuronavigation | n.g. | n.g. | Ki 67 | yes | n.g. | Not applicable | Measurement of PPIX, cell density | n.g. | no | Correlation with MET PET |
| Belloch et al. Acta Neurochir (Wien). 2014;156(4):653-60^5^ | Video imaging (exoscope)  ALA 20 mg/kg 2.5 -3 h prior to anesthesia | Retrospecitve (?), no PSP | 14 GBM, 1 grade II, 2 oligo grade III, 3 metastasis, 1 AA | n.g. | 21 | 14 in 7 patients (?) | n.g. | n.g. | n.g. | n.g. | n.g. | n.g. | n.g. | n.g. | n.g. | n.g. | Subjective scale from 1 to 5 (5 being the highest intensity). | no |  |
| Bongetta et al, World Neurosurg. 2016;88:54-8^6^ | Visual, fluorescein 20%, 5 mg/kg of sodium fluorescein at 20% (Monico S.p.a., Mestre, Italy) are administered intravenously at anesthesia induction | n.g. | 4 GBM | n.g. | 4 | n.g. | n.g. | n.g. | n.g | n.g. | n.g. | n.g. | n.g. | n.g. | n.g. | „Our results show a good distinction of fluorescein-stained structures” | n.g. | no |  |
| Bowden et al. Neurosurgery 82:719-727, 2018^7^ | Visual, 3 mg/kg fluorescein i.v. | n.g. | NE glioma | yes | 13 | 42 biopsies (“2-6 per patient”) | n.g. | n.g. | n.g. | n.g. | n.g. | yes | yes | n.g. | n.g. | n.g. | n.g. | no |  |
| Catapano et al. World Neurosurgery 104:239-247^8^ | Visual, fluorescein, 5 mg/kg at induction of anesthesia | Retrospective | Grade IV 22, grade III 1 | n.g. | 23 | n.g. | n.g. | n.g. | n.g. | n.g. | n.g. | n.g. | n.g. | n.g. | n.g. | Resection rates | n.g. | Case control n=25 |  |
| Chan et al. Asian J Surg  2018;41(5):467-472^9^ | Visual, ALA, 20 mg/kg, 2-4 hours prior to anesthesia | Retrospective | GBM 10  AA 2  DA 3  Others 1 | n.g. | 16 | n.g. | n.g. | n.g. | n.d. | n.g. | n.g. | n.g. | n.g. | yes | n.g. | Resection rates | n.g. | n.g. |  |
| Chen et al. Int J Med Sci. 2012;9(8):708-14^10^ | Visual, fluorescein, 10-20 mg intravenously after craniotomy | Prospective cohort, no PSP, group allocatio not described | 4 grade II, 3 AA, 3 GBM in fluorescein group, control group: 5 grade2, 3 AA, 4 GBM | n.g. | 22 | n.g. | n.g. | n.g. | n.g. | n.g. | n.g. | n.g. | n.g. | n.g. | n.g. | Gross total resection rate, PFS | n.g. | n.g. | No special microscope |
| Cortnum S, Dan Med J. 2012;59(8):A4460^11^ | Visual, ALA, 20mg/kg 2-4 h prior to surgery | Retrospective | 12 GBM, 1 PNET | no | 13 | n.g. | n.g. | n.g. | n.g. | n.g. | n.g. | n.g. | n.g. | n.g. | n.a. | Resection rates | n.g. | no |  |
| Della Puppa A, Biomed Res Int. 2014;2014:232561^12^ | Visual, ALA, 20mg/kg, 2-4h prior to surgery | Retrospective | HGG, 81 GBM, 13 grade III. | n.g., | 94 | n.g. | n.g. | n.g. | n.g. | n.g. | n.g. | n.g. | n.g. | n.g. | n.a. | n.g. | Resection rates | np |  |
| Della Puppa A et al. Acta Neurochir (Wien). 2013;155(6):965-72; discussion 72^13^ | Visual, ALA, 20mg/kg, 2-4h prior to surgery | Prospecttive, no PSP | HGG, 25 GBM, 4 grade III, 2 anaplastic oligodendroglioma | n.g. | 31 | n.g. | n.g. | n.g. | n.g. | n.g. | n.g. | n.g. | n.g. | n.g. | n.a. | Resection rates | neurological outcome | no |  |
| Ewelt et al. Clin Neurol Neurosurg. 2011;113(7):541-7^14^ | Visual, ALA, 20 mg/kg 2-4 h prior to indcution of anesthesia | Prospective, no PSP | 13 grade II, 15 grade III, 2 GBM | Yes, no ROC | 30 | 3 biopsies in 1 patient , 2 biopsies in 7 patients, 1 biopsy in 22 patiens | yes | no | Yes, neuronavigation | no | n.g. | n.g. | yes | no | n.e. | n.g. | n.g. | n.g. |  |
| Eyupoglu IY et al. Sci Rep. 2015;5:7958^15^ | 20 mg ALA/kg, 3 h prior to surgery, ICG 5ml of a 5mg/ml solution after ALA guided resection | Prospecitive, no PSP) | GBM | n.g. | 3 | 3 per patient | yes | yes | n.g. | n.g. | n.g. | yes | Yes,  No patient based assessment | n.g. | n.a. | n.g. | n.g. | no | Combination ALA and ICG after ALA resection |
| Eyupoglu IY PLoS One. 2012;7(9):e44885^16^ | Visual + imaging, ALA 20,g/kg 3 h prior to induction of anesthesia | Prospective, no PSP | 4 OA III, 3 AA, 30 GBM | n.g. | 37 | n.g. | n.g. | n.g. | n.g. | n.g. | n.g. | n.g. | n.g. | n.g. | n.a. | n.g. for imaging (Contrast-enhancing tumor not further elucidate) | Rates of resection | safety | Dual visualization technique,  ALA followed by intra-OP MRI |
| Floeth et al. Eur J Nucl Med Mol Imaging. 2011;38(4):731-41^17^ | Visual ALA 20mg/kg 2-4 hours prior to surgery | Prospective, no PSP | 19 grade III, 2 GBM, 11 grade II | yes | 30 | 38 biopsies, 6 patients 2 biopsies, 3 biopsies in 1 patient, 23 patients 1 biopsy | yes | n.g. | yes | n.g. | no | n.g. | yes | n.g. | Yes (FET) | n.g. | n.g. | n.g. |  |
| Francaviglia N, Surg Neurol Int. 2017;8:145^18^ | Visual, fluorescein 5 mg/kg immediately after induction of anethesia | Retrospective | 14 AA, 33 GBM | n.g. | 47 | n.g. | n.g. | n.g. | n.g. | n.g. | n.g. | n.g. | n.g. | n.g. | no | Rates of resection | “helpful” , “not helpful” | no | Comparison ALA, intraop MR |
| Gessler et al. Neurosurgery. 2015;77(1):16-22^19^ | visual 20 mg ALA/kg, 4-6 hours before surgery, imaging intraop MRI | Propsective | HGG, 27 GBM (new or rec), 5 grade III | Yes, no ROC | 32 | 38, per patient unknown | n.g. | n.g. | n.g. | n.g. | n.g. | n.g. | yes | n.g. | n.a. | Frequency residual tumor vs each modaility | n.g. | No, comparison in single patients |  |
| Hamamciolgu MK et al. Clinical Neurology and Neurosurgery 143 (2016) 39–45^20^ | Visual, fluorescein 2.4mg/kg after induction of anesthesaia | Retrospective | 15 grade IV, 6 grade III,2 lymphomas, 7 metastasis | n.g. | 29 | n.g. | n.g. | n.g. | n.g. | n.g. | n.g. | n.g. | n.g. | n.g. | n.g. | “helpful” “not helpful” | Resection rates | no |  |
| Hauser SB et al. Neurosurgery 78:475–483, 2016^21^ | Visual ALA 20mg/kg 4 hours prior to anesthesia, intraop MRI | Retrospective | 13 GBM, 1 lymphoma | yes | 14 | 117 | n.g. | n.g. | n.g. | n.g. | n.g. | yes | Yes, no patient based assessment | n.g. | Not given for MR CE | OS | n.g. | no |  |
| Hefti et al. Swiss Med Wkly. 2008;138(11-12):180-5^22^ | Visual, ALA 20mg/KG 5-6 hours prior to surgery |  | 47 GBM, 5 AA, 4 DA, q ependymoma, 14 other | Yes, no ROC | 74 | 62 bisopsies in 74 patients | n.g. | n.g. | n.g. | n.g. | n.g. | n.g. | yes | n.g. | n.a. | n.g. | n.g. | no |  |
| Hickmann AK et al. J Neurooncol. 2015;122(1):151-60^23^ | Visual, ALA , dose timing n.g. | Retrospective, no PSP | Recurrent glioma, 38 grade III, 25 grade IV | n.g. | 58 | n.g. | n.g. | n.g. | n.g. | n.g. | n.g. | n.g. | n.g. | n.g. | n.a. | PFS, OS | n.g. | no |  |
| Hong J et al. , Curr Probl Cancer. 2018^24^ | Visual, Fluorescein, 1.5-2 mg with anesthesia | Retrospective | HGG, 24 grade III, 18 grade IV | yes | 42 | 87 samples | n.g. | n.g. | n.g. | n.g. | n.g. | n.g. | Yes, no patient based assessment | n.g. | No, not for fluorescein, not for imaging | Volume of hemorrhage, duration of surgery, completeness of resection | n.g. | No, control group retrospective, not defined |  |
| Idoate et al. Neuropathology 2011 31:575-582^25^ | Visual, ALA, 20 mg/kg, 2-4 hours prior to induction of anesthesia | Retrospective, no PSP | GBM | n.g. | 30 | 152/30 | n.g. | yes | n.g. | n.g. | n.g. | yes | Yes, no patient based assessment | n.g. | n.g. | n.g. | n.g. | no |  |
|  |  |  |  |  |  |  |  |  |  |  |  |  |  |  |  |  |  |  |  |
| Jaber et al. Neurosurgery. 2018^26^ | Visual, ALA, 20 mg/kg, 3-4 hours prior to induction of anesthesia | Retrospective, no PSP | DA grade II | no | 74 | n.g. | n.g. | n.g. | n.g. | n.g. | n.g. | yes | n.g. | n.g. | Yes (PET) | PFS, Time to malig, degeneration, OS | n.g. | Control group wo fluorescence |  |
| Jaber et al. Neurosurgery. 2016;78(3):401-11^27^ | Visual 5.-ALA, 20mg/kg 3-4 hours prior to induction of anesthesia | Retrospective, no PSP | GBM 8, AA 76, DA 82 | Yes (FET PET for predicting fluorescdence) | 166 | n.g. | n.g. | n.g. | n.g. | n.g. | n.g. | n.g. | n.g. | yes | n.g. | n.g. | n.g. | no |  |
| Kuroiwa T et al. Neurol Res. 1999;21(1):130-4^28^ | Visual, fluorescein, 8mg/kg after incision of dura | Retrospective | “malignant glioma | n.g. | 5 | n.g. | n.g. | n.g. | n.g. | n.g. | n.g. | n.g. | n.g. | n.g. | n.g. | n.g. | Presence of tumor in fluorescing biopsies | no | no quantative data given |
|  |  |  |  |  |  |  |  |  |  |  |  |  |  |  |  |  |  |  |  |
| Kuroiwa et al. Surg Neurol. 1998;50(1):41-8^29^ | Visual, fluorescein,8mg/kg after incision of dura | Retrospective, no PSP | 5 grade III, 5 grade IV | n.g. | n.g. | n.g. | n.g. | n.g. | n.g. | n.g. | n.g. | n.g. | n.g. | n.g. | n.g. | OS | “reamrkable enhancement” | no |  |
| Lau D et al., J Neurosurg. 2016;124(5):1300-9^30^ | Visual, 5-ALA, 20mg/kg 3 hours prior to surgery | Prospective | Malignant glioma, 12 grade III, 47 grade IV | yes | 59 | 211 overall | No  „at least 1 biopsy from nonfluorescent tissue and at least 1 biopsy from the most fluorescent area” | n.g. | n.g. | n.g. | n.g. | n.g. | yes | no | n.a. | n.g. | n.g. | n.g. |  |
| Lee JY et al. Neurosurgery. 2016 December ; 79(6): 856–871^31^ | Imaging, ICG second window, 24 h prior to surgery 5 mg/kg | Prospective, no PSP | 2 grade 1, 2 grade 2, 1 grade III, 10 grade IV | yes | 15 | 71 overall | n.g. | n.g. | n.g. | n.g. | n.g. | n.g. | Yes; not evaluated on patient basis | n.g. | No, despite background signal of brain | Resection rates | n.g. | no |  |
| Liu et al. Chin Med J (Engl). 2013;126(13):2418-23^32^ | Visual, fluorescein, 1% 5ml after exposure of tumor | n.g. | 45 Grade I and II, 38 grade III grade IV | n.e. | 56 | n.g. | n.g. | n.g. | n.g. | n.g. | n.g. | n.g. | n.g. | n.g. | n.g. | Resection rates | Efficacy was assessed by comparisons of the tumor resection rate, the rate of induced paralysis during surgery, and the KPS score | no | No information on how control group was chosen, control group 27 patients |
| Moiyadi et al. J Neurol Surg A Cent Eur Neurosurg. 2014;75(6):434-41^33^ | Visual, ALA 20 mg/kg 4-6 hours prior to surgery | n.g. | 5 GBM, 3 AA | n.g. | 8 | n.g. | n.g. | n.g. | n.g. | n.g. | n.g. | n.g. | n.g. | n.g. | n.a. | Resection rates | Observance of fluorescence, outcome | no | Combination ALA, ioUS |
| Moiyadi et al. Neurol India. 2018;66(4):1087-93^34^ | Visual, ALA 20 mg/kg 4-6 hours prior to surgery | Retrospective, no PSP | 44 GBM, 6 grade III | Yes, no ROC | 50 | 29 from 12 patients | n.g. | n.g. | n.g. | n.g. | n.g. | n.g. | yes | n.g. | n.a. | Resection rates | n.g. | no |  |
| Nabavi et al. Neurosurgery. 2009;65(6):1070-6^35^ | Visual, ALA 20mg/kg 2.5-3.5 hours prior to induction of anesthesia | Prospective, multicentric | Recurrent, 21 GBM, 9 AA | yes |  | 354 from 30 patients | yes | yes | n.g. | n.g. | yes | yes | Yes, patient and biopsy based evaluation | no | n.a. | Resection rates | Neurological condition | no |  |
| Neira et al. **J Neurosurg** 127:111–122, 2016^36^ | Visual and imaging, fluorescein, 3 mg/kg follow- ing induction of anesthesia and prior to surgical incision. | Prospecitve, no PSP | GBM | Yes, based on image processing | 32 | 90 from 26 patients | n.g. | n.g. | yes | n.g. | n.g. | n.g. | Yes; no patient based assessment | n.g. | Not for MR; 10% for fluorescein video image, not for visual assessment | Resection rates | n.g. | Control group for resection |  |
| Ng WP et al. Malays J Med Sci. 2017;24(2):78-86^37^ | visual, ALA 20mg/kg, 3 h (range 2-4 h) | Retrospective | HGG 30 GBM, 5 AA, 2 others | n.g. | 37 ALA,  Control 37 | n.g. | n.g. | n.g. | n.g. | n.g. | n.g. | n.g. | n.g. | n.g. | n.a. | OS;, resection rates | safety | no | No information on control selection |
| Panciani et al. Clin Neurol Neurosurg. 2012;114(1):37-41^38^ | visual, ALA 20mg/kg, 3 h (range 2-4 h) | Prospective cohort | GBM | yes | 23 | 92 (4 per patient) | yes | yes | yes | no | n.g. | yes | Yes, no patient based evaluation | n.g. | n.a. | n.g. | n.g. | no |  |
| 38. Pettersen M et al. Photodiagnosis Photodyn Ther. 2014;11(3):351-6^39^ | visual, ALA 20mg/kg, 3 h (range 2-4 h) | n.g. | n.g. | n.g. | n.g. | n.g. | n.g. | n.g. | n.g. | n.g. | n.g. | n.g. | n.g. | n.g. | n.g. | n.g. | n.g. | n.g. | Study investigates perception of fluorescence on imaging |
| Piquer J et al. Biomed Res Int. 2014;2014:207974^40^ | visual, ALA 20mg/kg 5-6 hours prior to surgery | n.g. | HGG 23 GBM, 4 AA | yes | 27 | “5-7 biopsies per patient | n.g. | n.g. | n.g. | n.g. | n.g. | n.g. | yes | n.g. | n.a. | n.g. | n.g. | no | Evaluation exoscope |
| Rapp M et al. World Neurosurg. 2014;82(1-2):e277-9^41^ | Endoscopy, video imaging, ALA 20 mg/kg 3 h prior to surgery | Retrospective | 6 GBM, 1 radionecrosis, 1 metastasis, 1 ganglioblioma grade 1 | n.g. | 9 | 1 | no | n.g. | n.g. | n.g. | n.g. | n.g. | n.g. | n.g. | n.g. | n.g. | n.g. | n.g. |  |
| Rey-Dios R et al. Acta Neurochir (Wien). 2014;156(6):1071-5^42^ | Visual, fluorescein, 3mg/kg after induction of anesthesia | n.g. | 6 GBM | yes | 6 | 26, 3-5 samples per patient | n.g. | n.g. | yes | n.g. | n.g. | n.g. | Yes, not accounted for | n.g. | n.g. | n.g. | n.g. | no | Fluorescein used during stereotaxy |
| Ritz R, Eur J Surg Oncol. 2012;38(4):352-60^43^ | Visual, hypericin, 0.1 mg/kg body weight) 6 h before the surgical procedure |  | Recurrent malignant glioma | yes | 5 | 110 | n.g. | n.g. | n.g. | n.g. | n.g. | n.g. | Yes, no patient based evaluation | n.g. | No threshold given | resection | n.g. | no |  |
| Roberts DW J Neurosurg. 2011;114(3):595-603^44^ | Visual, ALA 20mg/kg 3 h prior to induction of anesthesia | Prospective, no PSP | GBM | yes | 11 | 124 | n.g. | yes | yes | n.g. | Yes; Mixed models for random effects | n.g. | Yes;no patient based assessment | n.g. | MR CE quantified | n.g. | n.g. | no |  |
| Schebesch KM et al. Clin Neurol Neurosurg. 2018;172:177-82^45^ | Visual, fluorescein, 5 mg/kg 10% at induction of anesthesia | Retrospective | 1 grade 1, 1 grade II, 3 grade III | n.g. | 5 | Not given | Form fluorescing tissue (?) | yes | yes | n.g. | n.g. | yes | n.g. | n.g. | No data given for PET nor intra-operative fluorescein | n.g. | n.g. | no |  |
| Shah et al. Asian J Neurosurg. 2016;11(3):276-81^46^ | ICG visualization of vessel | n.a. | n.a. | n.a. | n.a. | n.a. | n.a. | n.a. | n.a. | n.a. | n.a. | n.a. | n.a. | n.a. | n.a. | n.a. | n.a. | n.a. | ICG visualization of vessels in insular glioma |
| Stummer W et al. Lancet Oncol. 2006;7(5):392-401^47^ | Visual, ALA, 20 mg/kg 3 hours (range 2-4 hours) prior to induction of anesthesia | Prospective, multicentric, randomized, PSP | Malignant glioma, 237 GBM, 33 AA | n.g. | 270 patients, 139 ALA, 131 WL | n.e. | n.g. | n.g. | n.g. | n.g. | n.g. | n.g. | n.g. | n.g. | n.a. | Resection rates, PFS; OS | safety | randomized |  |
| Stummer W et al. Neurosurgery. 2017;81(2):230-9^48^ | Visual, ALA, 20 mg/kg 3 hours (range 2-4 hours) prior to induction of anesthesia | Prospective, single centerdouble blinded trial, PSP | 1 grade III,  20 grade IV | n.g. | 21 | 154, 7 per patient | n.g. | n.g. | n.g. | n.g. | n.g. | n.g. | Yes, no patient base assessment | n.g. | Spectrography with threshold definition | Fluorescence ratios, cell densities | no | Control groups | Randomized 3 dose study |
| Stummer et al. Neurosurgery. 1998;42(3):518-25;^49^ | Visual, ALA, 10 mg/kg 3 hours prior to induction of anesthesia | Prospcetive single arm, no PSP | 2 AA, 8GBM | yes | 10 | 89 overall, 5-16 biopsies/patient | n.g. | n.g. | n.g. | n.g. | n.g. | n.g. | Yes, not accounted for | n.g. | n.a. | n.g. | n.g. | no |  |
| Stummer et al. 2014 Neurosurgery 74: 310-320^50^ | Visual, ALA, 10 mg/kg 3 hours prior to induction of anesthesia | Prospcetive single arm, PSP | 4 grade II, 29 grade IV | yes | 33 | 300 | yes | yes | yes | n.g. | Same frequency per patient | yes | Yes  Patient based assessment | n.g. | n.a. | n.g. | n.g. | no |  |
| Szmuda et al. J Neurooncol. 2015;122(3):575-84^51^ | Visual, ALA, 20 mg/kg 4 hours prior to induction of anesthesia | Prospective | 2 grade II, 19 grade IV | n.g. | 21 | n.g. | n.g. | n.g. | n.g. | n.g. | n.g. | n.g. | n.g. | n.g. | n.a. | n.g. | n.g. | no | Correlation of human perception with video image |
| Tsugu et al. World Neurosurg. 2011;76(1-2):120-7^52^ | Visual, 5-ALA 1 g 2 hours (range 1-3 h) prior to induction of anesthesia | Retrospecitve | 6 grade II, 7 grade III, 20 grade IV | n.g. | 33 in two grops (MR, ALA) | n.g. | n.g. | n.g. | n.g. | n.g. | n.g. | n.g. | n.g. | n.g. | MR as comparatornot well defined | Resection rates | n.g. | no | Comparison resection rates MR,ALA |
| Utsuki et al. Neurol Med Chir (Tokyo). 2008;48(2):95-7^53^ | Spectrgraphical tumor detector, 1g ALA 2 hours before anesthesia | prospective | n.g. | n.g. | n.g. | n.a | n.a. | n.a. | n.a. | n.a. | n.a. | n.a. | n.a. | no | yes | n.g. | n.g. | no | Technical note testing new detector |
| Valdes et al. J Neurosurg. 2015;123(3):771-80^54^ | Visual and spectrographic, 5-ALA, 20 mg/kg 3 hours prior to induction of anesthesia | Prospective. PSP: not given | LGG | yes | 12 | Overall 73 (1-11) | n.g. | n.g. | n.g. | n.g. | Yes;  Mixed models for random effects | n.g. | Yes; no patient based assessment | n.g. | Defined For quantitative spectrography: | n.g. | n.g. | no |  |
| Valdes et al. Neuro Oncol. 2011;13(8):846-56^55^ | Spectorgraphy and visual, ALA 20 mg/kg 3 hour prior to anesthesia | Prospective, PSP n.g. | 7 LGG, 3 AA, 13 GBM | n.g. | 23 | 133 overall | n.g. | n.g. | n.g. | n.g. | yes | yes | yes | n.g. | Threshold for spectrographical C(PPIX) defined | n.g. | n.g. | no |  |
| Valdes et al. J Biomed Opt. 2011;16(11):116007^56^ | Spectrography and reflectance, ALA, | Prospective, no PSP | 2 LGG, 8 HGG | yes | 10 | 88 overall | n.g. | n.g. | n.g. | n.g. | n.g. | n.g. | yes | n.g. | Yes for spectography, reflectance | n.g. | n.g. | no |  |
| Widhalm et al. PLoS One. 2013;8(10):e76988^57^ | Visual, ALA, 20mg/kg 3 h prior to anesthesia | Prospective cohort, no PSP | Grade II 33, grade III 26 | yes | 59 | n.g. | From fluorescing tissue, if encountered | yes | yes | n.g. | Only one value counted per patient | n.g. | n.g. | n.g. | Yes, FET PET | n.g. | n.g. | no | evaluates the predictive accuracy of any fluorescence for schowing anaplasia |
| Widhalm et al. Cancer. 2010;116(6):1545-52^58^ | Visual, ALA 20mg/kg 3 hours prior to anesthesia | Prospective cohort, no PSP | 8 grade II, 9 grade III | n.g. | 17 | n.g. | From fluorescing tissue, if encounterd | yes | yes | n.g. | Only one value counted per patient | n.g. | n.g. | n.g. | Yes, FET PET | n.g. | n.g. | no |  |
| Xiang J et al. Br J Neurosurg. 2018;32(2):141-8^59^ | Visual, fluorescein, 5mg/kg after induction of anesthesia | n.g., no PSP | 23 HGG, 5 LGG | n.g. | 28 | n.g. | n.g. | n.g. | n.g. | n.g. | n.g. | n.g. | n.g. | n.g. | n.g. | Resection rates, survival | Fluorescence yes/ no vs. global histology | no |  |
| Yamada S et al. Clin Neurol Neurosurg. 2015;130:134-9^60^ | Visual and MR,5-ALA 20mg/kg, 2-4h prior to induction of anesthesia | Retrospective | 33 grade III, 67 grade IV | yes | 99 | 286, 1-10 per patient | n.g. | n.g. | n.g. | n.g. | n.g. | n.g. | Yes | n.g. | n.g. | n.g. | n.g. | no |  |
| Zeh R et al. 2017, PLoS ONE 12(7): e0182034 ^61^ | Imaging, ICG second window, 24 h prior to surgery 5 mg/kg | Prospective | GBM | yes | 10 | 15 biopsies | n.g. | n.g. | n.g. | n.g. | n.g. | n.g. | Yes; no patient based evaluation | yes | Threshold not given despite background signal | n.g. | n.g. | no |  |
|  |  |  |  |  |  |  |  |  |  |  |  |  |  |  |  |  |  |  |  |
| Zhang N et al. Biomed Res Int. 2017;2017:7865747^62^ | Visual, fluorescein, 2-3 mg /kg after induction of anesthesia | Retrospective | 10 grade III, 28 IV | yes | 38 | 89 overall, 1-8 per patient | n.g. | n.g. | n.g. | n.g. | n.g. | n.g. | yes | n.g. | n.g. | OS; PFS | n.g. | no |  |

**References**

1. Acerbi F, Broggi M, Broggi G, Ferroli P: What is the best timing for fluorescein injection during surgical removal of high-grade gliomas? **Acta Neurochir (Wien) 157:**1377-1378, 2015

2. Acerbi F, Broggi M, Eoli M, Anghileri E, Cuppini L, Pollo B, et al: Fluorescein-guided surgery for grade IV gliomas with a dedicated filter on the surgical microscope: preliminary results in 12 cases. **Acta Neurochir (Wien) 155:**1277-1286, 2013

3. Acerbi F, Broggi M, Schebesch KM, Hohne J, Cavallo C, De Laurentis C, et al: Fluorescein-Guided Surgery for Resection of High-Grade Gliomas: A Multicentric Prospective Phase II Study (FLUOGLIO). **Clin Cancer Res 24:**52-61, 2018

4. Arita H, Kinoshita M, Kagawa N, Fujimoto Y, Kishima H, Hashimoto N, et al: (1)(1)C-methionine uptake and intraoperative 5-aminolevulinic acid-induced fluorescence as separate index markers of cell density in glioma: a stereotactic image-histological analysis. **Cancer 118:**1619-1627, 2012

5. Belloch JP, Rovira V, Llacer JL, Riesgo PA, Cremades A: Fluorescence-guided surgery in high grade gliomas using an exoscope system. **Acta Neurochir (Wien) 156:**653-660, 2014

6. Bongetta D, Zoia C, Pugliese R, Adinolfi D, Silvani V, Gaetani P: Low-Cost Fluorescein Detection System for High-Grade Glioma Surgery. **World Neurosurg 88:**54-58, 2016

7. Bowden SG, Neira JA, Gill BJA, Ung TH, Englander ZK, Zanazzi G, et al: Sodium Fluorescein Facilitates Guided Sampling of Diagnostic Tumor Tissue in Nonenhancing Gliomas. **Neurosurgery 82:**719-727, 2018

8. Catapano G, Sgulo FG, Seneca V, Lepore G, Columbano L, di Nuzzo G: Fluorescein-Guided Surgery for High-Grade Glioma Resection: An Intraoperative "Contrast-Enhancer". **World Neurosurg 104:**239-247, 2017

9. Chan DTM, Yi-Pin Sonia H, Poon WS: 5-Aminolevulinic acid fluorescence guided resection of malignant glioma: Hong Kong experience. **Asian J Surg 41:**467-472, 2018

10. Chen B, Wang H, Ge P, Zhao J, Li W, Gu H, et al: Gross total resection of glioma with the intraoperative fluorescence-guidance of fluorescein sodium. **Int J Med Sci 9:**708-714, 2012

11. Cortnum S, Laursen RJ: Fluorescence-guided resection of gliomas. **Dan Med J 59:**A4460, 2012

12. Della Puppa A, Ciccarino P, Lombardi G, Rolma G, Cecchin D, Rossetto M: 5-Aminolevulinic acid fluorescence in high grade glioma surgery: surgical outcome, intraoperative findings, and fluorescence patterns. **Biomed Res Int 2014:**232561, 2014

13. Della Puppa A, De Pellegrin S, d'Avella E, Gioffre G, Rossetto M, Gerardi A, et al: 5-aminolevulinic acid (5-ALA) fluorescence guided surgery of high-grade gliomas in eloquent areas assisted by functional mapping. Our experience and review of the literature. **Acta Neurochir (Wien) 155:**965-972; discussion 972, 2013

14. Ewelt C, Floeth FW, Felsberg J, Steiger HJ, Sabel M, Langen KJ, et al: Finding the anaplastic focus in diffuse gliomas: the value of Gd-DTPA enhanced MRI, FET-PET, and intraoperative, ALA-derived tissue fluorescence. **Clin Neurol Neurosurg 113:**541-547, 2011

15. Eyupoglu IY, Hore N, Fan Z, Buslei R, Merkel A, Buchfelder M, et al: Intraoperative vascular DIVA surgery reveals angiogenic hotspots in tumor zones of malignant gliomas. **Sci Rep 5:**7958, 2015

16. Eyupoglu IY, Hore N, Savaskan NE, Grummich P, Roessler K, Buchfelder M, et al: Improving the extent of malignant glioma resection by dual intraoperative visualization approach. **PLoS One 7:**e44885, 2012

17. Floeth FW, Sabel M, Ewelt C, Stummer W, Felsberg J, Reifenberger G, et al: Comparison of (18)F-FET PET and 5-ALA fluorescence in cerebral gliomas. **Eur J Nucl Med Mol Imaging 38:**731-741, 2011

18. Francaviglia N, Iacopino DG, Costantino G, Villa A, Impallaria P, Meli F, et al: Fluorescein for resection of high-grade gliomas: A safety study control in a single center and review of the literature. **Surg Neurol Int 8:**145, 2017

19. Gessler F, Forster MT, Duetzmann S, Mittelbronn M, Hattingen E, Franz K, et al: Combination of Intraoperative Magnetic Resonance Imaging and Intraoperative Fluorescence to Enhance the Resection of Contrast Enhancing Gliomas. **Neurosurgery 77:**16-22; discussion 22, 2015

20. Hamamcioglu MK, Akcakaya MO, Goker B, Kasimcan MO, Kiris T: The use of the YELLOW 560 nm surgical microscope filter for sodium fluorescein-guided resection of brain tumors: Our preliminary results in a series of 28 patients. **Clin Neurol Neurosurg 143:**39-45, 2016

21. Hauser SB, Kockro RA, Actor B, Sarnthein J, Bernays RL: Combining 5-Aminolevulinic Acid Fluorescence and Intraoperative Magnetic Resonance Imaging in Glioblastoma Surgery: A Histology-Based Evaluation. **Neurosurgery 78:**475-483, 2016

22. Hefti M, von Campe G, Moschopulos M, Siegner A, Looser H, Landolt H: 5-aminolevulinic acid induced protoporphyrin IX fluorescence in high-grade glioma surgery: a one-year experience at a single institutuion. **Swiss Med Wkly 138:**180-185, 2008

23. Hickmann AK, Nadji-Ohl M, Hopf NJ: Feasibility of fluorescence-guided resection of recurrent gliomas using five-aminolevulinic acid: retrospective analysis of surgical and neurological outcome in 58 patients. **J Neurooncol 122:**151-160, 2015

24. Hong J, Chen B, Yao X, Yang Y: Outcome comparisons of high-grade glioma resection with or without fluorescein sodium-guidance. **Curr Probl Cancer**, 2018

25. Idoate MA, Diez Valle R, Echeveste J, Tejada S: Pathological characterization of the glioblastoma border as shown during surgery using 5-aminolevulinic acid-induced fluorescence. **Neuropathology 31:**575-582, 2011

26. Jaber M, Ewelt C, Wolfer J, Brokinkel B, Thomas C, Hasselblatt M, et al: Is Visible Aminolevulinic Acid-Induced Fluorescence an Independent Biomarker for Prognosis in Histologically Confirmed (World Health Organization 2016) Low-Grade Gliomas? **Neurosurgery**, 2018

27. Jaber M, Wolfer J, Ewelt C, Holling M, Hasselblatt M, Niederstadt T, et al: The Value of 5-Aminolevulinic Acid in Low-grade Gliomas and High-grade Gliomas Lacking Glioblastoma Imaging Features: An Analysis Based on Fluorescence, Magnetic Resonance Imaging, 18F-Fluoroethyl Tyrosine Positron Emission Tomography, and Tumor Molecular Factors. **Neurosurgery 78:**401-411; discussion 411, 2016

28. Kuroiwa T, Kajimoto Y, Ohta T: Comparison between operative findings on malignant glioma by a fluorescein surgical microscopy and histological findings. **Neurol Res 21:**130-134, 1999

29. Kuroiwa T, Kajimoto Y, Ohta T: Development of a fluorescein operative microscope for use during malignant glioma surgery: a technical note and preliminary report. **Surg Neurol 50:**41-48; discussion 48-49, 1998

30. Lau D, Hervey-Jumper SL, Chang S, Molinaro AM, McDermott MW, Phillips JJ, et al: A prospective Phase II clinical trial of 5-aminolevulinic acid to assess the correlation of intraoperative fluorescence intensity and degree of histologic cellularity during resection of high-grade gliomas. **J Neurosurg 124:**1300-1309, 2016

31. Lee JY, Thawani JP, Pierce J, Zeh R, Martinez-Lage M, Chanin M, et al: Intraoperative Near-Infrared Optical Imaging Can Localize Gadolinium-Enhancing Gliomas During Surgery. **Neurosurgery 79:**856-871, 2016

32. Liu JG, Yang SF, Liu YH, Wang X, Mao Q: Magnetic resonance diffusion tensor imaging with fluorescein sodium dyeing for surgery of gliomas in brain motor functional areas. **Chin Med J (Engl) 126:**2418-2423, 2013

33. Moiyadi A, Shetty P: Navigable intraoperative ultrasound and fluorescence-guided resections are complementary in resection control of malignant gliomas: one size does not fit all. **J Neurol Surg A Cent Eur Neurosurg 75:**434-441, 2014

34. Moiyadi AV, Sridhar E, Shetty P, Madhugiri VS, Solanki S: What you see and what you don't - Utility and pitfalls during fluorescence guided resections of gliomas using 5-aminolevulinic acid. **Neurol India 66:**1087-1093, 2018

35. Nabavi A, Thurm H, Zountsas B, Pietsch T, Lanfermann H, Pichlmeier U, et al: Five-aminolevulinic acid for fluorescence-guided resection of recurrent malignant gliomas: a phase ii study. **Neurosurgery 65:**1070-1076; discussion 1076-1077, 2009

36. Neira JA, Ung TH, Sims JS, Malone HR, Chow DS, Samanamud JL, et al: Aggressive resection at the infiltrative margins of glioblastoma facilitated by intraoperative fluorescein guidance. **J Neurosurg:**1-12, 2016

37. Ng WP, Liew BS, Idris Z, Rosman AK: Fluorescence-Guided versus Conventional Surgical Resection of High Grade Glioma: A Single-Centre, 7-Year, Comparative Effectiveness Study. **Malays J Med Sci 24:**78-86, 2017

38. Panciani PP, Fontanella M, Schatlo B, Garbossa D, Agnoletti A, Ducati A, et al: Fluorescence and image guided resection in high grade glioma. **Clin Neurol Neurosurg 114:**37-41, 2012

39. Petterssen M, Eljamel S, Eljamel S: Protoporphyrin-IX fluorescence guided surgical resection in high-grade gliomas: The potential impact of human colour perception. **Photodiagnosis Photodyn Ther 11:**351-356, 2014

40. Piquer J, Llacer JL, Rovira V, Riesgo P, Rodriguez R, Cremades A: Fluorescence-guided surgery and biopsy in gliomas with an exoscope system. **Biomed Res Int 2014:**207974, 2014

41. Rapp M, Kamp M, Steiger HJ, Sabel M: Endoscopic-assisted visualization of 5-aminolevulinic acid-induced fluorescence in malignant glioma surgery: a technical note. **World Neurosurg 82:**e277-279, 2014

42. Rey-Dios R, Hattab EM, Cohen-Gadol AA: Use of intraoperative fluorescein sodium fluorescence to improve the accuracy of tissue diagnosis during stereotactic needle biopsy of high-grade gliomas. **Acta Neurochir (Wien) 156:**1071-1075; discussion 1075, 2014

43. Ritz R, Daniels R, Noell S, Feigl GC, Schmidt V, Bornemann A, et al: Hypericin for visualization of high grade gliomas: first clinical experience. **Eur J Surg Oncol 38:**352-360, 2012

44. Roberts DW, Valdes PA, Harris BT, Fontaine KM, Hartov A, Fan X, et al: Coregistered fluorescence-enhanced tumor resection of malignant glioma: relationships between delta-aminolevulinic acid-induced protoporphyrin IX fluorescence, magnetic resonance imaging enhancement, and neuropathological parameters. Clinical article. **J Neurosurg 114:**595-603, 2011

45. Schebesch KM, Brawanski A, Doenitz C, Rosengarth K, Proescholdt M, Riemenschneider MJ, et al: Fluorescence-guidance in non-Gadolinium enhancing, but FET-PET positive gliomas. **Clin Neurol Neurosurg 172:**177-182, 2018

46. Shah A, Rangarajan V, Kaswa A, Jain S, Goel A: Indocyanine green as an adjunct for resection of insular gliomas. **Asian J Neurosurg 11:**276-281, 2016

47. Stummer W, Pichlmeier U, Meinel T, Wiestler OD, Zanella F, Reulen HJ, et al: Fluorescence-guided surgery with 5-aminolevulinic acid for resection of malignant glioma: a randomised controlled multicentre phase III trial. **Lancet Oncol 7:**392-401, 2006

48. Stummer W, Stepp H, Wiestler OD, Pichlmeier U: Randomized, Prospective Double-Blinded Study Comparing 3 Different Doses of 5-Aminolevulinic Acid for Fluorescence-Guided Resections of Malignant Gliomas. **Neurosurgery 81:**230-239, 2017

49. Stummer W, Stocker S, Wagner S, Stepp H, Fritsch C, Goetz C, et al: Intraoperative detection of malignant gliomas by 5-aminolevulinic acid-induced porphyrin fluorescence. **Neurosurgery 42:**518-525; discussion 525-516, 1998

50. Stummer W, Tonn JC, Goetz C, Ullrich W, Stepp H, Bink A, et al: 5-Aminolevulinic acid-derived tumor fluorescence: the diagnostic accuracy of visible fluorescence qualities as corroborated by spectrometry and histology and postoperative imaging. **Neurosurgery 74:**310-319; discussion 319-320, 2014

51. Szmuda T, Sloniewski P, Olijewski W, Springer J, Waszak PM: Colour contrasting between tissues predicts the resection in 5-aminolevulinic acid-guided surgery of malignant gliomas. **J Neurooncol 122:**575-584, 2015

52. Tsugu A, Ishizaka H, Mizokami Y, Osada T, Baba T, Yoshiyama M, et al: Impact of the combination of 5-aminolevulinic acid-induced fluorescence with intraoperative magnetic resonance imaging-guided surgery for glioma. **World Neurosurg 76:**120-127, 2011

53. Utsuki S, Oka H, Miyajima Y, Shimizu S, Suzuki S, Fujii K: Auditory alert system for fluorescence-guided resection of gliomas. **Neurol Med Chir (Tokyo) 48:**95-97; discussion 97-98, 2008

54. Valdes PA, Jacobs V, Harris BT, Wilson BC, Leblond F, Paulsen KD, et al: Quantitative fluorescence using 5-aminolevulinic acid-induced protoporphyrin IX biomarker as a surgical adjunct in low-grade glioma surgery. **J Neurosurg 123:**771-780, 2015

55. Valdes PA, Kim A, Brantsch M, Niu C, Moses ZB, Tosteson TD, et al: delta-aminolevulinic acid-induced protoporphyrin IX concentration correlates with histopathologic markers of malignancy in human gliomas: the need for quantitative fluorescence-guided resection to identify regions of increasing malignancy. **Neuro Oncol 13:**846-856, 2011

56. Valdes PA, Kim A, Leblond F, Conde OM, Harris BT, Paulsen KD, et al: Combined fluorescence and reflectance spectroscopy for in vivo quantification of cancer biomarkers in low- and high-grade glioma surgery. **J Biomed Opt 16:**116007, 2011

57. Widhalm G, Kiesel B, Woehrer A, Traub-Weidinger T, Preusser M, Marosi C, et al: 5-Aminolevulinic acid induced fluorescence is a powerful intraoperative marker for precise histopathological grading of gliomas with non-significant contrast-enhancement. **PLoS One 8:**e76988, 2013

58. Widhalm G, Wolfsberger S, Minchev G, Woehrer A, Krssak M, Czech T, et al: 5-Aminolevulinic acid is a promising marker for detection of anaplastic foci in diffusely infiltrating gliomas with nonsignificant contrast enhancement. **Cancer 116:**1545-1552, 2010

59. Xiang Y, Zhu XP, Zhao JN, Huang GH, Tang JH, Chen HR, et al: Blood-Brain Barrier Disruption, Sodium Fluorescein, And Fluorescence-Guided Surgery Of Gliomas. **Br J Neurosurg 32:**141-148, 2018

60. Yamada S, Muragaki Y, Maruyama T, Komori T, Okada Y: Role of neurochemical navigation with 5-aminolevulinic acid during intraoperative MRI-guided resection of intracranial malignant gliomas. **Clin Neurol Neurosurg 130:**134-139, 2015

61. Zeh R, Sheikh S, Xia L, Pierce J, Newton A, Predina J, et al: The second window ICG technique demonstrates a broad plateau period for near infrared fluorescence tumor contrast in glioblastoma. **PLoS One 12:**e0182034, 2017

62. Zhang N, Tian H, Huang D, Meng X, Guo W, Wang C, et al: Sodium Fluorescein-Guided Resection under the YELLOW 560 nm Surgical Microscope Filter in Malignant Gliomas: Our First 38 Cases Experience. **Biomed Res Int 2017:**7865747, 2017
