# Supplementary material for: Intraoperative fluorescence diagnosis in the brain: a systematic review and suggestions for future standards on reporting diagnostic accuracy and clinical utility
Source: Acta Neurochir (Wien). 2019 Jul 30;161(10):2083–98. doi: 10.1007/s00701-019-04007-y (PMC6739423; doi:10.1007/s00701-019-04007-y)
Supplement: Supplementary file 2 — (DOCX 20 kb) [file 701_2019_4007_MOESM2_ESM.docx]

**Appendix 2:**

**Recommendations pertaining to statistical analysis and handling of dependent and clustered samples:**

The performance of a diagnostic test is often expressed in terms of sensitivity and specificity compared to a reference standard (standard of truth). Calculations of sensitivity and specificity may involve multiple observations per patient, which implies that the data are clustered. Whether analysis of sensitivity and specificity per patient or using multiple observations per patient is preferable generally depends on the clinical context and consequences.

In the present context, calculations of sensitivity and specificity are not valid in the sense of a normal diagnostic test.

One particular bias results from the aspect that the intra-operative signal (e.g. fluorescence) will influence the regions in which biopsies are collected and that the surgical exposure of the tumor will naturally also centered on the tumor, and surgery will be in the region of the gross tumor. Thus, the decision of where to sample in the brain is not independent of tumor location but depends on the exposure, which is usually limited to the tumor, and the visible signal. For an unbiased calculation of sensitivity and specificity, however, histology would have to be independent of the anatomical exposure and the optical signal. Hypothetically, if all samples were taken in the highlighted tumor center, the sensitivity would even be equal 100% and the specificity 0%.

The analysis should focus on the PPV and NPV. Both values depend on the prevalence of tumor cells, which, in an infiltrating tumor, is directly related to biopsy location.

The following covariates and variables should be recorded and reported:

- location of the biopsy (center, margin, normal brain, distance of biopsy from tumor), optimally as pre-defined by MRI. The distance and the location are directly related to varying prevalence.
- time point after application of the diagnostic agent at which the biopsy is taken.
- if possible, the number of biopsies per patient should be equal and pre-specified
- multiple observations per patient are relevant to the clinical decision problem, so the potential correlation between observations should be explored and taken into account in the statistical analysis. An analysis ignoring correlations between multiple biopsies within one patient often yields misleadingly small estimated standard errors and 95% confidence intervals [CI]) for the sensitivity and specificity because all observations are erroneously counted as independent observations. Therefore, the use of generalized linear mixed models (GLMMs)^2,6^ as appropriate statistical method is recommended. GLMMs should be used to estimate the adjusted PPV and NPV, respectively. The true value (dichotomous pathology result) should be the dependent variable. The optical signal (dichotomous/continuous) and the necessary covariates should be included as fixed effects. To account for the clustered data structure (multiple biopsies from one patient), a random intercept for the patient should be included.
- The detailed model specification should be reported to enhance study transparency, e.g. included fixed effects, interaction terms, random effects, fitted covariance structures and estimation methods.
- If the optical signal gives a continuous measure, it should be clearly reported how the influence on the PPV or NPV was modeled (transformations, linear, quadratic, cubic, etc.), respectively.
- Additionally, receiver operating characteristic (ROC) and predictive receiver operating characteristic (PROC) analysis could be performed.
- Other common methods to adjust sensitivity and specificity for clustered data are simple CI adjustments (ratio estimator)^4,8^ or variance inflation factor^1^, or generalized estimating equations (GEEs)^3,5,7^. Nevertheless, these methods are less appropriate in terms of covariable adjustment and missing values.
- Comparison of different diagnostic methods can be performed within the GLMMs. Randomization of the patients to the diagnostic methods is recommended.
- The number of patients and biopsies per patient should be reported. All results should be reported as estimates with corresponding confidence limits.
- A biopsy-based analysis should be the main point of interest. Additionally, a patient-based analysis can be performed as sensitivity analysis. Averaging single biopsies from a single patient in a particular region is not recommended, because of the inherent loss of information.
- If possible, blinded evaluations concerning different methods of tissue diagnosis should be performed. Likewise, the pathologists should be blinded.

**Appendix References**

1. Fleiss JL, Levin B, Paik M: **Statistical methods for rates and proportions, 3rd edition**: Wiley, 2003

2. Karim MR, Zeger SL: Generalized linear models with random effects; salamander mating revisited. **Biometrics 48:**631-644, 1992

3. Leisenring W, Pepe MS, Longton G: A marginal regression modelling framework for evaluating medical diagnostic tests. **Stat Med 16:**1263-1281, 1997

4. Rao JN, Scott AJ: A simple method for the analysis of clustered binary data. **Biometrics 48:**577-585, 1992

5. Smith PJ, Hadgu A: Sensitivity and specificity for correlated observations. **Stat Med 11:**1503-1509, 1992

6. Verbeke G, Molenberghs G: **Linear Mixed Models for Longitudinal Data**: Springer, 2000

7. Zeger SL, Liang KY: Longitudinal data analysis for discrete and continuous outcomes. **Biometrics 42:**121-130, 1986

8. Zhou X, Obuchowski N, McClish D: **Statistical Methods in Diagnostic Medicine, 2nd Edition**, 2002
